# A CT imaging-based prediction model of functional outcome and benefit of endovascular thrombectomy for ischemic stroke

**DOI:** 10.1007/s00330-025-12207-7

**Published:** 2026-01-14

**Authors:** Sven PR Luijten, Aravind Ganesh, Adam P. Marcus, Paul Bentley, Daniel Rueckert, Scott Brown, Faysal Benali, Joachim Fladt, Fouzi Bala, Ibrahim Alhabli, Keith W. Muir, Jeffrey Saver, Andrew M. Demchuk, Tudor G. Jovin, Serge Bracard, Bruce CV Campbell, Francis Guillemin, Philip White, Michael D. Hill, Peter J. Mitchell, Charles BLM Majoie, Mayank Goyal, Diederik WJ Dippel, Aad van der Lugt, Theo van Walsum, Hester F. Lingsma, Daniel Bos

**Affiliations:** 1 Department of Radiology & Nuclear Medicine, Erasmus MC University Medical Center, Rotterdam, The Netherlands; 2 Calgary Stroke Program, Departments of Clinical Neurosciences and Community Health Sciences, Hotchkiss Brain Institute and the O’Brien Institute for Public Health, Cumming School of Medicine, University of Calgary, Calgary, AB Canada; 3 Division of Brain Sciences, Department of Medicine, Imperial College London, London, UK; 4 BioMedIA Group, Department of Computing, Imperial College London, London, UK; 5 Klinikum Rechts der Isar, Technical University of Munich, Munich, Germany; 6 Altair Biostatistics, St Louis Park, MN USA; 7 Department of Radiology, Maastricht University Medical Center, Maastricht, The Netherlands; 8 Department of Radiology, AZ Vesalius, Tongeren, Belgium; 9 Diagnostic and Interventional Neuroradiology Department, University Hospital of Tours, Tours, France; 10 Institute of Neuroscience and Psychology, University of Glasgow, Queen Elizabeth University Hospital, Glasgow, UK; 11 Department of Neurology, David Geffen School of Medicine at the University of California, Los Angeles, CA USA; 12 Stroke Institute, Department of Neurology, University of Pittsburgh Medical Center, Pittsburgh, PA USA; 13 Department of Diagnostic and Interventional Neuroradiology, INSERM U 1254, Université de Lorraine, University Hospital of Nancy, Nancy, France; 14 Department of Medicine and Neurology, Melbourne Brain Centre at the Royal Melbourne Hospital, University of Melbourne, Parkville, VIC Australia; 15 Clinical Investigation Centre - Clinical Epidemiology, INSERM 1433, Université de Lorraine, University Hospital of Nancy, Nancy, France; 16 Translational and Clinical Research Institute, Faculty of Medical Sciences, Newcastle University and Newcastle upon Tyne Hospitals NHS Trust, Newcastle, UK; 17 Department of Radiology, Royal Melbourne Hospital, University of Melbourne, Parkville, VIC Australia; 18 Department of Radiology and Nuclear Medicine, Amsterdam University Medical Centers, University of Amsterdam, Amsterdam, The Netherlands; 19 Calgary Stroke Program, Departments of Clinical Neurosciences and Radiology, Hotchkiss Brain Institute, Cumming School of Medicine, University of Calgary, Calgary, AB Canada; 20 Department of Neurology, Erasmus University Medical Center, Rotterdam, The Netherlands; 21 Department of Public Health, Erasmus University Medical Center, Rotterdam, The Netherlands; 22 Department of Epidemiology, Erasmus University Medical Center, Rotterdam, The Netherlands

**Keywords:** Ischemic stroke, Thrombectomy, Computed tomography.

## Abstract

**Objective:**

To investigate the value of baseline CT imaging for the prediction of functional outcome and benefit of endovascular thrombectomy (EVT) for anterior large vessel occlusion (LVO).

**Materials and methods:**

We used individual patient data from seven randomized EVT trials and included patients with available baseline CT imaging and outcome data. We developed a model to predict functional outcome and benefit of EVT, including baseline stroke-related and brain frailty CT imaging features alone. We compared the discriminative performance of our model for predicting good functional outcome (modified Rankin Scale [mRS] 0–2) and treatment benefit (difference between the probability of mRS 0–2 with vs without EVT) with MR PREDICTS by calculating the difference in *C*-statistics (delta C and delta C-for-benefit).

**Results:**

We included 1391 patients (median age, 67 years, interquartile range 59–76; 53% male). Discrimination of the model based on CT imaging alone was substantial for the prediction of good functional outcome (*C*-statistic 0.700, 95% CI: 0.666–0.731) and treatment benefit (C-for-benefit 0.640, 95% CI: 0.586–0.690). After adding the known strongest clinical predictors namely age and National Institutes of Health Stroke Scale score, discrimination improved to slightly lower than MR PREDICTS for prediction of good functional outcome (*C*-statistic 0.733 vs 0.750; delta C, −0.017 [95% CI: −0.037 to 0.003]) and treatment benefit (C-for-benefit 0.675 vs 0.692; delta C-for-benefit −0.017 [95% CI: −0.084 to 0.050]).

**Conclusions:**

Baseline CT imaging holds considerable predictive value with regard to functional outcome and treatment benefit, but a combination of clinical and imaging features offers the best predictive performance.

**Key Points:**

***Question*** *The predictive value of baseline CT imaging for the prediction of functional outcome and benefit of EVT for anterior LVO stroke is uncertain*.

***Findings*** *Discrimination of a model based on CT imaging alone is substantial, but can further be improved by the addition of limited clinical characteristics*.

***Clinical relevance*** *Baseline CT imaging holds considerable predictive value with regard to functional outcome and treatment benefit. The addition of limited clinical information is needed to achieve predictive performance similar to an established prediction model*.

**Graphical Abstract:**

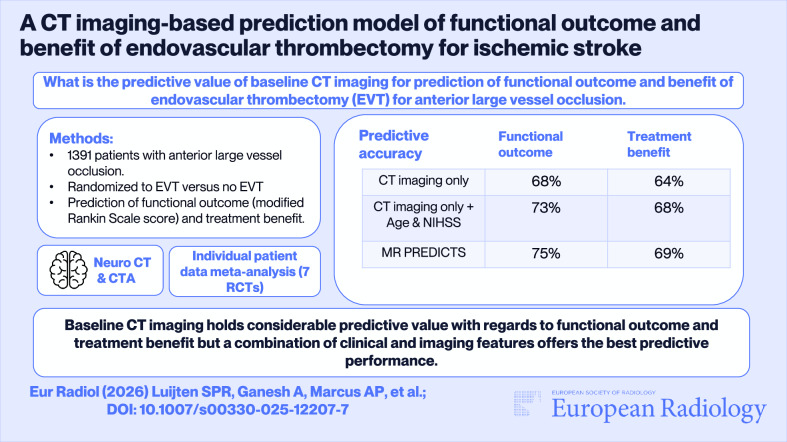

## Introduction

Despite the large beneficial effect of endovascular thrombectomy (EVT) on functional outcome for groups of patients with anterior large vessel occlusion (LVO) ischemic stroke enrolled in landmark randomized clinical trials (RCTs)^1^, individual patient benefit varies substantially. Approximately half of all patients undergoing EVT did not achieve functional independence after EVT, underlining the need to better identify patients most likely to benefit from EVT and prevent futile treatment [1].

In most countries, baseline CT imaging, including non-contrast CT and CT angiography, is the cornerstone of the diagnostic work-up of ischemic stroke. In addition to its use in diagnosis, baseline CT imaging potentially also holds important information regarding patient prognosis and the individual patient's benefit of EVT. Numerous prediction models have been developed incorporating stroke-related imaging features linked to functional outcome, and thereby also hold information on potential benefit from EVT [2]. This includes Alberta Stroke Program Early CT Score (ASPECTS), occlusion location, and collateral score [3]. Interestingly, imaging features of pre-existent subclinical brain frailty, such as white matter lesions (WML), brain atrophy, old infarcts, and intracranial carotid artery calcification (ICAC), have until now not been considered, but were all associated with adverse outcomes after EVT for ischemic stroke [4–7]. These brain frailty imaging features may provide unique additional information for predicting functional outcome and benefit of EVT.

As CT imaging is well implemented in routine diagnostic pathways for ischemic stroke patients, using this information for outcome prediction is highly feasible and requires only a single data source. Furthermore, image processing methods are being developed to extract prognostic imaging features in a quantitative and automated manner. In contrast, existing prediction models such as MR PREDICTS require stroke physicians to gather information regarding patient medical history, laboratory measurements, workflow, and imaging at the time of EVT decision-making [2, 8].

Therefore, we aimed to investigate the predictive value of baseline CT imaging alone for the prediction of functional outcome and benefit of EVT. In addition, we sought to investigate the prognostic and predictive value of individual CT imaging features of subclinical brain frailty. To this end, we compared the performance of prediction models that included only CT imaging data with the MR PREDICTS model, which is based on a combination of clinical and imaging data.

## Materials and methods

We first developed and validated models for predicting functional outcome and benefit of EVT based on baseline CT imaging features alone, and then in combination with age and baseline National Institutes Health Stroke Scale (NIHSS) score. We compared the predictive performance of these models with the existing MR PREDICTS model as a reference. Second, we determined whether brain frailty imaging features provided unique added predictive value over the clinical characteristics and stroke-related imaging features incorporated in MR PREDICTS.

### Study design and participants

We used pooled data from the Highly Effective Reperfusion Evaluated in Multiple Endovascular Stroke trials (HERMES) collaboration, including individual patient data (*n* = 1764) from seven RCTs (MR CLEAN [9], ESCAPE [10], EXTEND-IA [11], SWIFT PRIME [12], REVASCAT [13], THRACE [14], and PISTE [15]). All seven trials compared the efficacy of EVT (intervention) vs standard care alone (control) in patients with ischemic stroke due to anterior LVO (*n* = 1764). For the present analysis, we used data of 1391 patients with outcome data and baseline CT imaging available, randomly allocated to the EVT (*n* = 691) or standard care group (*n* = 700; Supplementary Fig. 1). Written informed consent was provided by all patients according to each trial protocol, and each study was approved by the local ethics board [1].

### Outcome measures

**Fig. 1:**
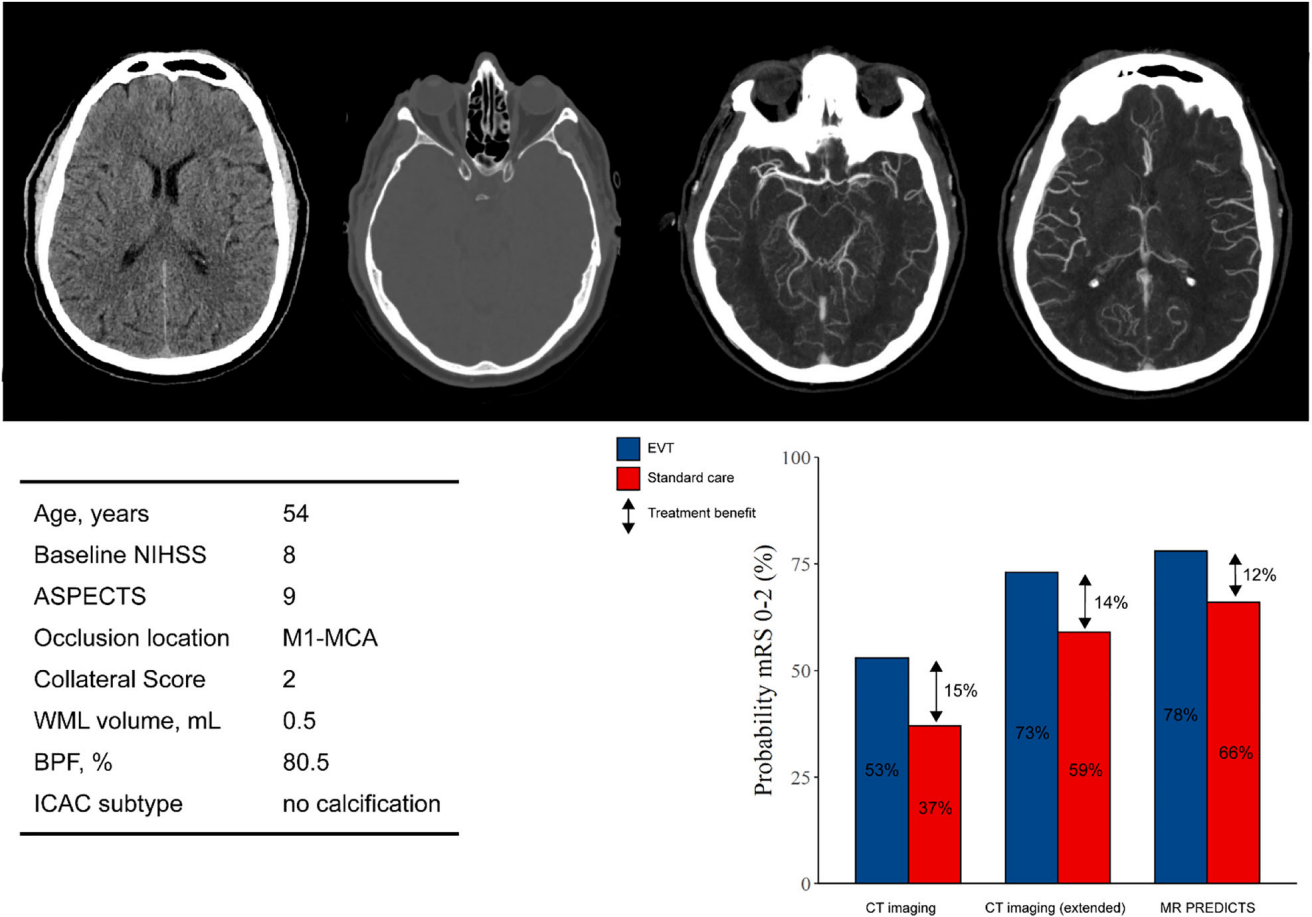
Example of prediction of good functional outcome (mRS score 0–2) and treatment benefit (difference in probability of mRS 0–2 with and without EVT) using different models

Functional outcome at 90 days after randomization was measured on the modified Rankin Scale (mRS), an ordinal scale ranging from 0 (no symptoms) to 6 (dead) [16]. The mRS was modeled as a full ordinal scale in the proposed CT imaging-based model, and MR PREDICTS, and we subsequently extracted probabilities of good functional outcome (mRS 0–2) from the predicted distribution (mRS 0–6). We defined treatment benefit as the difference between the probability of good functional outcome with vs without EVT (Fig. 1).

### Assessment of predictor variables

**Fig. 2:**
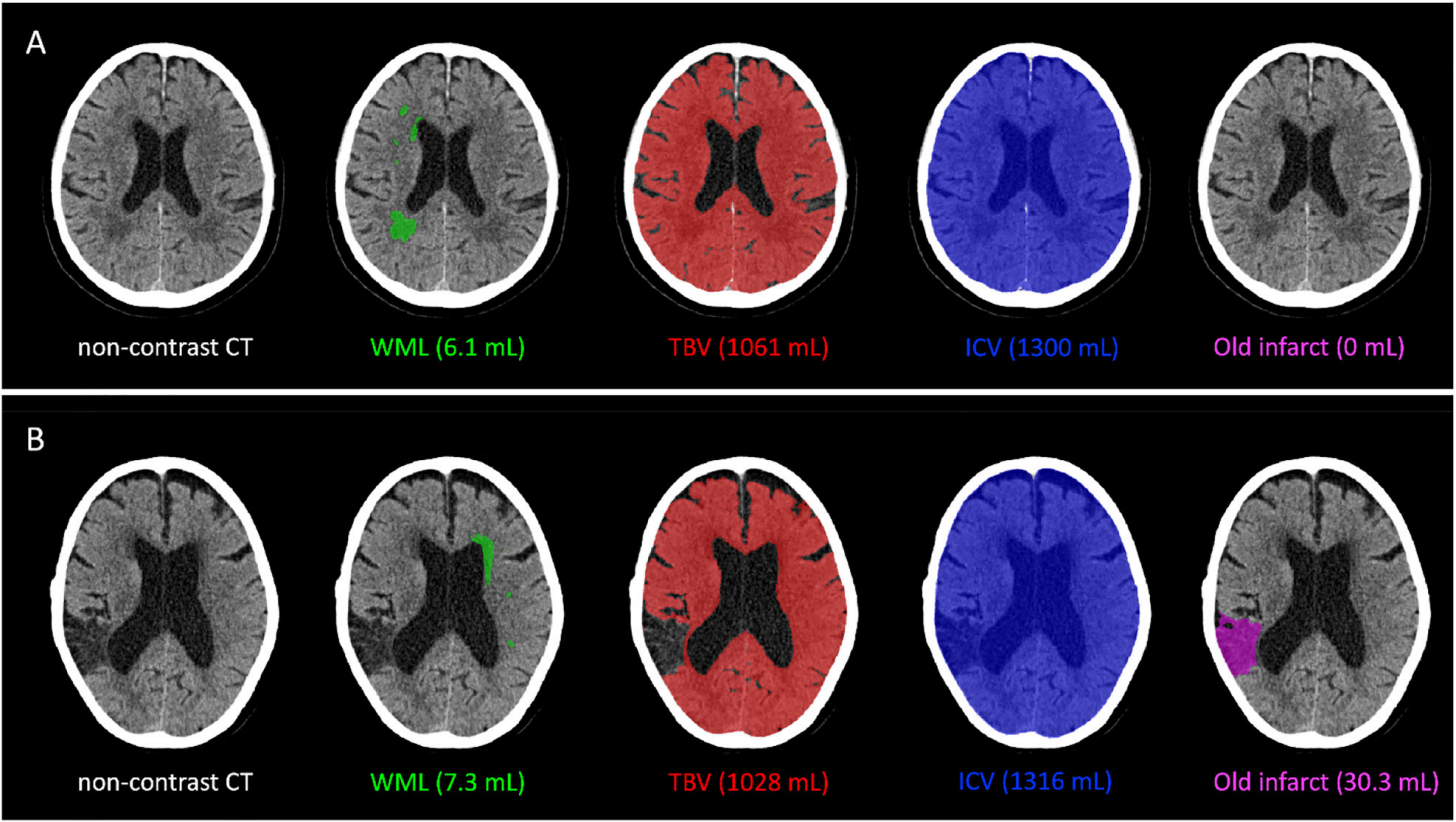
Examples of segmentations on baseline non-contrast CT in two individual patients. **A** Patient with a left M1 middle cerebral artery occlusion. **B** Patient with a right M1 middle cerebral artery occlusion. White matter lesion (WML) volume in the asymptomatic hemisphere is shown in green, total brain volume (TBV) is shown in red, and intracranial volume (ICV) is shown in blue. Brain atrophy was determined as the proportion of TBV in relation to ICV expressed as the brain parenchymal fraction (BPF, in %). Old infarct volume shown in magenta

We assessed WML, brain atrophy, old infarcts, and ICAC subtype on baseline non-contrast CT. WML volume, brain atrophy, and old infarct volumes were assessed quantitatively using automated segmentation algorithms (Fig. 2; details described in the supplements). WML and old infarct volumes were determined using a multiclass U-Net segmentation model based upon prior expert classification. This method automatically quantifies volumes of WML and old infarcts on non-contrast CT scans [17, 18]. We determined WML volume only in the contralateral hemisphere, because WML and early ischemic changes are both characterized by hypo-attenuation of brain tissue on CT, potentially resulting in overestimation of WML volume in the ipsilateral hemisphere. This is considered a valid approach due to the high interhemispheric correlation of WML burden [19]. We determined brain atrophy by measuring the proportion of total brain volume in relation to intracranial volume expressed as the brain parenchymal fraction (BPF, in %) using CTSeg. CTSeg is an extension of the unified segmentation algorithm for SPM12 with several enhancements, including improved registration, priors on the Gaussian mixture model parameters, and an atlas learned from both MRIs and CTs [20, 21]. Total brain volume was computed as the sum of gray matter (GM) and white matter (WM), and intracranial volume was computed as the sum of GM, WM, and cerebrospinal fluid (CSF). We assessed the presence of ICAC and ICAC subtypes in the intracranial carotid artery on the side of stroke using a previously validated visual scoring method. This method evaluates the circularity, thickness, and morphology of calcifications using a specific weighting, and ICAC subtype was categorized as no calcification, intimal ICAC, or medial ICAC [22]. Data for the number of scans assessed for each brain frailty variable are described in Supplementary Fig. 1.

Data on other predictor variables used for analyses have previously been collected within HERMES [1]. Data on stroke-related imaging variables, including ASPECTS, site of occlusion, and collateral score, have previously been assessed by independent HERMES core laboratories masked to all clinical information, except side of stroke [3].

### Statistical analysis

First, we assessed the association of brain frailty imaging features with functional outcome at 90 days. Non-linearity of WML volume, BPF, and old infarct volume was evaluated using restricted cubic splines. We also tested the potential effect modification of EVT by each brain frailty feature separately using multiplicative interaction terms. Interaction terms were only added to the CT imaging-based prediction model if these were statistically significant (*p* < 0.05) and biologically plausible, or on the basis of strong biological plausibility alone. This is because data-driven inclusion of interaction terms makes a model prone to overfitting and exaggerating the heterogeneity of the treatment effect [23].

Second, we constructed a multivariable ordinal prediction model including predictor variables derived solely from baseline non-contrast CT and CT angiography, including WML volume, BPF, old infarct volume, ICAC subtype, ASPECTS, site of occlusion, and collateral score. We then simplified this model through backward elimination, removing variables with a p-value of > 0.20 in multivariable analysis. We extended our imaging-based model with age and baseline NIHSS score, which are the known strongest predictors of functional outcome [24, 25]. We added age to test whether chronological age offers added predictive value over brain frailty as the measure of biological age.

Third, we assessed model performance through internal-external cross-validation using a leave-one-study-out approach [26]. In this way, the model is developed in all available RCTs except one, which is left out as a validation cohort. We repeated this process until each RCT within HERMES is left out once, resulting in seven estimates of model performance. This method ensures all available data is used for model development and allows evaluating cohort-specific effects, as well as the model’s generalizability. We assessed the discriminative performance of our model using Harrell’s *C*-statistic. For the prediction of ordinal outcomes, the *C*-statistic is computed by randomly selecting pairs of patients and evaluating whether the patient with the higher observed mRS score also has a higher predicted mRS score. In this way, the *C*-statistic measures the probability that in a randomly selected pair of patients, the one with the higher observed outcome level also has the higher predicted probability or score, in line with the ordering. For calibration, we constructed calibration plots displaying predicted vs observed probability of good functional outcome and quantified the calibration slope and intercept.

Fourth, we computed the C-for-benefit, a metric specifically developed to measure a model’s discriminative ability to predict treatment benefit [27]. This metric represents the probability that from two randomly chosen matched patient pairs with unequal observed treatment benefit, the pair with greater observed benefit also has a higher predicted benefit. It is important to note that the effects of two counterfactual treatment strategies, i.e., EVT vs standard of care, are inherently unobservable in individual patients. Therefore, we defined observed treatment benefit as the difference in probability of good functional outcome in pairs of patients matched on baseline characteristics but discordant for treatment allocation (1:1 ratio). We visually assessed the agreement between predicted vs observed treatment benefit using benefit calibration plots and quantified the difference between the average predicted vs average observed benefit defined as the mean calibration.

Fifth, we determined whether brain frailty provided added predictive value to MR PREDICTS which includes the following baseline clinical and imaging variables: age, NIHSS score, pre-stroke mRS, diabetes mellitus, systolic blood pressure, treatment with IV alteplase, blood glucose level, ASPECTS, site of occlusion, collateral score, and time from stroke onset to groin puncture [8, 28]. We sequentially added each brain frailty variable and evaluated whether model fit improved based on a likelihood ratio test (*p* < 0·05). In case of improved model fit, we also assessed whether model performance improved [29].

Finally, performance measures in the seven cross-validation sets were pooled using random-effects meta-analysis and reported as point estimates with corresponding 95% confidence intervals (CI). Differences in discriminative performance of the models were compared by calculating the difference in concordance statistics (delta C and delta C-for-benefit), and 95% CIs were obtained by using a bootstrapping procedure drawing 500 random samples with replacement.

Missing predictor variables were imputed using multiple imputation (n = 5). Results of the analyses are reported in accordance with the transparent reporting of a multivariable prediction model for individual patient prognosis or diagnosis (TRIPOD) guideline [30]. Statistical analyses were done in R (version 4.1.1.) with packages Hmisc, Rms, CalibrationCurves, HTEPredictionMetrics, meta, and forest.

## Results

At 90 days, 315/691 (45.6%) patients allocated to the EVT group and 195/700 (27.9%) allocated to the standard care group achieved a good functional outcome (Table 1). Patients with good functional outcome had a lower degree of brain atrophy, indicated by higher BPF, and were less likely to have WML, old infarcts, and ICAC (Table 1). Brain frailty characteristics were more prevalent at higher age; per quartile of advancing age, patients were more likely to have WML, old infarcts, and ICAC, the latter especially of the medial subtype (Supplementary Table 1). Furthermore, we observed a nonlinear increase in WML volume and a nonlinear decrease in BPF with advancing age (Supplementary Fig. 2).

**Table 1:** Patient characteristics stratified by good (mRS 0–2) vs poor (mRS 3–6) functional outcome

	Good functional outcome (*n* = 510)	Poor functional outcome (*n* = 881)
EVT	315 (61.8%)	376 (42.7%)
Age (years)	65 (54–74)	69 (59–78)
Male sex	274 (53.7%)	459 (52.1%)
Diabetes	64 (12.5%)	181 (20.6%)
Prior stroke	54 (10.6%)	104 (11.8%)
Pre-stroke mRS		
0	389 (76.3%)	614 (69.7%)
1	39 (7.6%)	117 (13.3%)
≥ 2	9 (1.8%)	52 (5.9%)
Baseline NIHSS	15 (11–19)	18 (15–21)
Systolic blood pressure (mmHg)	142 (22)	148 (26)
Glucose (mg/dL)	119 (104–145)	125 (110–154)
IV alteplase	451 (88.4%)	763 (86.6%)
Time between onset to groin puncture (min)	220 (165–285)	245 (195–302)
ASPECTS	9 [8–10]	9 [7–10]
Occlusion location		
ICA/ICA-T	71 (14.3%)	222 (25.5%)
M1	382 (76.7%)	596 (68.5%)
M2	45 (9.0%)	52 (6.0%)
Collateral score		
0—Absent	3 (0.6%)	11 (1.2%)
1—Poor	39 (7.6%)	156 (17.7%)
2—Moderate	199 (39.0%)	347 (39.4%)
3—Good	221 (43.3%)	283 (32.1%)
Any WML	375 (79.4%)	716 (86.9%)
WML volume (mL)	0.83 (0.40–2.03)	1.14 (0.45–3.07)
BPF (%)	80.69 (2.58)	80.18 (2.84)
Any old infarct	100 (21.1%)	219 (26.4%)
Old infarct volume (mL)	0.54 (0.10–3.45)	0.64 (0.15–6.86)
ICAC subtype		
No calcification	157 (32.0%)	188 (22.3%)
Intimal	164 (33.4%)	229 (27.1%)
Medial	170 (34.6%)	427 (50.6%)

Data are presented as count (%), mean (SD), or median (IQR)

*EVT* endovascular thrombectomy, *mRS* modified Rankin Scale, *NIHSS* National Institutes of Health Stroke Scale, *ASPECTS* Alberta Stroke program CT score, *BPF* brain parenchymal fraction, *WML* white matter lesion, *ICAC* intracranial carotid artery calcification

WML volume, BPF, and ICAC subtype were significant predictors of functional outcome in univariable (Supplementary table 2) and multivariable analysis (Table 2), but no significant effect of old infarct volume was found. The association between BPF and functional outcome was nonlinear in multivariable analysis. Therefore, we added BPF as a nonlinear predictor to the models and, to ease interpretation, present effect estimates separately above and below the inflection point of 80% (Table 2 and Supplementary Fig. 3). The final CT imaging-based model included ASPECTS, occlusion location, collateral score, WML volume, BPF, and ICAC subtype. In line with the development of MR PREDICTS, we included an interaction term between EVT and collateral score in our CT imaging-based model. Further addition of interaction terms was not considered as we found no evidence for effect modification of EVT by BPF (*p* = 0.64), WML volume (*p* = 0.64), or ICAC subtype (*p* = 0.73).

**Table 2:** Main predictors effects for each model in HERMES (*n* = 1391)

	CT imaging-based	CT imaging-based (extended)	MR PREDICTS (reference)
EVT	1.92 (1.59–2.32)	2.04 (1.69–2.47)	1.90 (1.52–2.33)
Age^a^			
Per year increase < 65		0.99 (0.98–1.00)	1.00 (0.98–1.01)
Per year increase ≥ 65		0.95 (0.93–0.96)	0.94 (0.93–0.96)
Baseline NIHSS, per point		0.94 (0.92–0.95)	0.93 (0.91–0.95)
Systolic blood pressure^a^			
Per 10 mmHg increase < 130			1.11 (0.96–1.28)
Per 10 mmHg increase ≥ 130			0.85 (0.81–0.90)
IV alteplase			1.04 (0.77–1.40)
Diabetes			0.71 (0.54–0.92)
Glucose^a^			
Per 10 mg/dL increase < 120			0.94 (0.86–1.03)
Per 10 mg/dL increase ≥ 120			0.98 (0.96–1.00)
Prestoke mRS			0.63 (0.55–0.76)
Time from stroke onset to groin puncture, per 30 min			0.92 (0.89–0.96)
ASPECTS, per point	1.17 (1.11–1.24)	1.17 (1.11–1.24)	1.17 (1.10–1.24)
Occlusion location			
ICA/ICA-T	1.00 (reference)	1.00 (reference)	1.00 (reference)
M1	1.70 (1.34–2.14)	1.56 (1.23–1.97)	1.63 (1.29–2.07)
M2	2.84 (1.86–4.34)	2.14 (1.39–3.28)	2.29 (1.58–3.79)
Collateral score	1.60 (1.40–1.83)	1.45 (1.26–1.66)	1.44 (1.26–1.67)
WML volume, per 5 mL	0.80 (0.73–0.90)	0.88 (0.79–0.98)	
BPF^a^			
Per 1% decrease < 80%	0.90 (0.83–0.98)	0.89 (0.83–0.98)	
Per 1% decrease ≥ 80%	0.96 (0.90–1.03)	0.97 (0.91–1.04)	
Old infarct volume, per 5 mL	1.00 (0.92–1.08)	0.99 (0.93–1.07)	
ICAC subtype			
No calcification	1.00 (reference)	1.00 (reference)	
Intimal ICAC	0.92 (0.71–1.19)	1.10 (0.84–1.46)	
Medial ICAC	0.51 (0.40–0.66)	0.78 (0.58–1.04)	

All effect estimates represent common odds ratios with 95% CIs, where an odds ratio > 1 corresponds with a better functional outcome

*EVT* endovascular thrombectomy, *NIHSS* National Institutes of Health Stroke Scale, *mRS* modified Rankin Scale, *ASPECTS* Alberta Stroke program early CT score, *BPF* brain parenchymal fraction, *WML* white matter lesion, *ICAC* intracranial carotid artery calcification

^a^ Age, systolic blood pressure, glucose, and BPF are modeled using restricted cubic spline functions. For Age, systolic blood pressure, and glucose, odds ratios are given for an increase below and above the respective inflection points. For BPF, odds ratios are given for a decrease below and above the inflection point

**Fig. 3:**
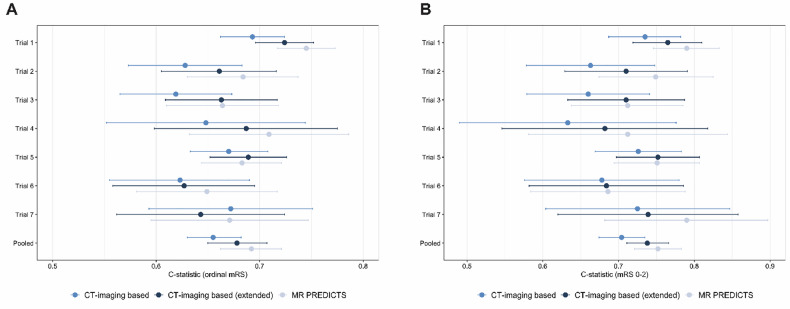
Plot showing results of the internal-external cross-validation using a leave-one-study-out approach. *C*-statistics with corresponding 95% confidence intervals of different models per individual trial and pooled estimates of all 7 cross-validation sets estimated using random effects meta-analysis for (**A**) prediction of ordinal mRS and for (**B**) prediction of good functional outcome (mRS 0–2)

At internal-external cross-validation, the pooled *C*-statistic of the CT imaging-based model was 0.656 (95% CI: 0.630–0.682) for prediction of ordinal mRS and 0.700 (95% CI: 0.666–0.731) for prediction of good functional outcome (Table 3). MR PREDICTS showed higher discrimination compared to our CT imaging-based model (*C*-statistic 0.750; delta C, −0.050 [95% CI: −0.078 to 0.022]). After extending the CT imaging-based model with age and baseline NIHSS, the *C*-statistic improved to 0.678 (95% CI: 0.650–0.707) for the prediction of ordinal mRS and to 0.733 (95% CI: 0.699–0.763) for the prediction of good functional outcome. Discrimination of the extended CT imaging-based model was slightly lower compared to the MR PREDICTS for the prediction of good functional outcome (delta C, −0.017 [95% CI: −0.037 to 0.003]) (Fig. 3).

**Table 3:** Performance estimates for the prediction of functional outcome and treatment benefit for different models

	CT imaging-based^a^	CT imaging-based^b^ (extended)	MR PREDICTS^c^ (reference)
Functional outcome			
*C*-statistic (ordinal)	0.656 (0.630–0.682)	0.678 (0.650–0.707)	0.692 (0.662–0.721)
*C*-statistic (mRS 0–2)	0.700 (0.666–0.731)	0.733 (0.699–0.763)	0.750 (0.718–0.781)
Calibration slope	1.059 (0.891–1.228)	0.994 (0.847–1.141)	0.989 (0.840–1.138)
Calibration intercept	0.276 (−0.131 to 0.684)	0.274 (−0.142 to 0.691)	0.273 (−0.112 to 0.659)
Treatment benefit			
C-for-benefit	0.640 (0.586–0.690)	0.675 (0.627–0.721)	0.692 (0.638–0.741)
Mean calibration	0.014 (−0.050 to 0.077)	0.020 (−0.047 to 0.088)	0.010 (−0.057 to 0.076)

Pooled estimates with corresponding 95% confidence intervals in parentheses were calculated using random-effects meta-analysis

*mRS* modified Rankin scale, *BPF* brain parenchymal fraction, *WML* white matter lesion, *ICAC* intracranial carotid artery calcification, *ASPECTS* Alberta stroke program early CT score, *NIHSS* National Institutes of Health Stroke Scale

^a^ Includes BPF, WML volume, ICAC subtype, ASPECTS, occlusion location, and collateral score

^b^ Includes age, baseline NIHSS, BPF, WML volume, ICAC subtype, ASPECTS, occlusion location, and collateral score

^c^ Includes age, baseline NIHSS, systolic blood pressure, IV alteplase, diabetes, glucose, pre-stroke mRS, time from stroke onset to groin puncture, ASPECTS, occlusion location, and collateral score

Agreement between the predicted and observed outcomes was comparable for all models (Table 3). Pooled estimates indicated consistent miscalibration, with a lower proportion of patients predicted to achieve a good functional outcome than observed. However, there was some heterogeneity between individual trials (Supplementary Figs. 4–6).

The pooled C-for-benefit of our CT imaging-based model was 0.640 (95% CI: 0.586–0.690) for the prediction of treatment benefit, which was lower compared to MR PREDICTS (C-for-benefit 0.640 vs 0.692; delta C-for-benefit −0.052 [95% CI: −0.099 to 0.005]). After extending the CT imaging-based model with age and baseline NIHSS, the C-for-benefit improved to 0.675 (95% CI: 0.627–0.721) and was slightly lower compared to MR PREDICTS (delta C-for-benefit −0.017 [95% CI: −0.084 to 0.050]). Agreement between average predicted and observed treatment was comparable for all models (Table 3 and Supplementary Figs. 7–9).

The addition of WML volume (*p* = 0.03), BPF (*p* = 0.01), and ICAC subtype (*p* = 0.03) to MR PREDICTS significantly improved model fit. However, pooled discriminative performance of the extended model remained comparable to the reference MR PREDICTS model for prediction of outcome (*C*-statistic 0.748 vs 0.750, delta C −0.002 [95% CI: −0.013 to 0.008]) and benefit of EVT (C-for-benefit 0.692 vs 0.692; delta C 0.000 [95% CI: −0.069 to 0.066]).

## Discussion

Discrimination of our CT imaging-based model was substantial for the prediction of functional outcome and benefit of EVT, but lower than MR PREDICTS. After extending the CT imaging-based model with age and baseline NIHSS, the predictive accuracy improved and was only slightly lower than MR PREDICTS. Incorporating brain frailty features in MR PREDICTS improved model fit but did not provide added predictive value in terms of improved discriminative ability.

Our CT imaging-based model provided lower predictive performance than MR PREDICTS for the prediction of functional outcome and benefit of EVT, but included only 6 variables that are all derived from a single data source, i.e., baseline CT imaging, which is almost always acquired when CT is the primary imaging modality in suspected acute LVO stroke. In contrast, MR PREDICTS incorporates 11 variables, including information from medical records, laboratory measurements, workflow, and baseline CT imaging [8, 28]. This may limit the clinical utility of this model, as extensive information regarding patient medical history, laboratory measurements, workflow, and baseline CT imaging needs to be gathered at the time of EVT decision-making. After extending our CT imaging-based model with limited clinical information of just age and baseline NIHSS, model discrimination improved and was only slightly lower than MR PREDICTS. Both age and baseline NIHSS can usually be obtained prior to or very shortly after performing baseline CT imaging, making these variables feasible to use as additional inputs for the proposed CT imaging-based prediction model. Further improvements of our CT imaging-based model could potentially also be provided by adding CT perfusion (CTP) parameters together with information regarding the eloquence of affected brain regions [31, 32]. However, since CTP was available in only 34% of patients pooled within HERMES, the addition of CTP parameters to our CT imaging-based model was not considered in the current study [3]. Additionally, since CTP is less widely used compared to non-contrast CT and CTA, adding CTP parameters would reduce the generalizability of the model.

Some variation in discrimination and calibration was seen when different trials were held out for validation. Besides differences in true predictor effects and chance, this heterogeneity in model performance reflects differences in case-mix and baseline probability of good functional outcome between individual trials, resulting mainly from varying patient selection criteria. In general, trials with more inclusive selection criteria have a larger case-mix variation, making it easier for a model to distinguish between low- and high-risk patients indicated by a higher *C*-statistic. Likewise, in the present study, variation observed in miscalibration, especially the intercept, reflects differences in the proportion of patients achieving a good functional outcome between trials within HERMES, which ranged from 26% to 56%.

Even though the addition of brain frailty variables to MR PREDICTS improved model fit assessed using likelihood ratio tests, discriminative ability remained unchanged. Therefore, it seems that brain frailty imaging features evaluated here do not provide incremental prognostic value compared to clinical and stroke-related imaging variables incorporated in MR PREDICTS. Identifying additional baseline factors that provide substantial additional predictive value over factors already incorporated in MR PREDICTS is a challenging task. This is because functional outcome is not only determined by factors known at admission, but also by (post)-procedural factors occurring over the course of time after the decision to perform EVT has been made and prior to functional outcome assessment at 90 days [33]. Most importantly, the quality of reperfusion and occurrence of adverse events such as pneumonia and symptomatic intracranial hemorrhage [24, 25]. Yet, as the current model is intended to be used at the time of decision-making to perform EVT, this information is simply unknown.

The proposed CT imaging-based model requires the output of different segmentation algorithms. However, the algorithms we used here are not developed for clinical use in the acute stroke setting and can therefore, unlike MR PREDICTS, not yet be used in routine clinical practice. More work would first be needed to develop and implement these methods in acute stroke imaging workflows as has already been done for software tools providing automated real-time assessment of ASPECTS, occlusion detection, and collateral score. Exploring deep-learning-based approaches could also be valuable, particularly end-to-end deep learning-based methods using raw CT imaging data as input without prior human-involved feature extraction [34, 35]. Future work, preferably with access to larger and more recent datasets, should determine the potential merits and feasibility of such efforts.

The strengths of this study include using a large cohort of patients randomized to EVT vs standard care, pooled from seven RCTs. Additionally, we used automated segmentation methods to quantitatively determine brain atrophy, WML burden, and extent of old infarcts. By doing so, we determined the effects of these variables on a continuous scale instead of a discrete scale when using visual assessment methods such as the Fazekas scale for WML or the Global Cortical Atrophy scale for brain atrophy. The use of these discrete scales is generally considered disadvantageous for prediction as it requires using arbitrary cutoff points to stratify patients, thereby resulting in potential loss of important predictive information [36]. Limitations of our study must also be considered. We developed and validated our CT imaging-based model using baseline CT scans. Therefore, the model is not directly applicable to patients in whom baseline imaging is performed with MRI. Nevertheless, CT is the preferred imaging modality for diagnosis in patients with suspected LVO stroke in most institutions. Additionally, ICAC is easily assessable on non-contrast CT but not on sequences included in routine MRI protocols used for patients with suspected stroke. Second, we used data of patients who were recruited according to selective trial criteria, especially regarding pre-stroke mRS, baseline NIHSS, ASPECTS, and collateral status. Thus, the patients included in the present analysis do not fully reflect the totality of ischemic stroke patients that are encountered in routine clinical practice. In addition, general practice today is likely not comparable to the period when the RCTs within HERMES were performed due to the development of new devices, new treatment techniques, more experienced (neuro)interventionalists, and better recanalization rates. Therefore, performance metrics of currently evaluated prediction models are likely not generalizable to patient subgroups routinely excluded from RCTs within HERMES or to general practice today. Specifically, to patients with pre-stroke disability, low NIHSS, and less favorable imaging profiles, e.g., indicated by low ASPECTS and poor collaterals. Although the latter patient group could be subject to additional predictive model refinement based on developing individual-patient data meta-analysis of large core trials.

We demonstrated that baseline CT imaging holds considerable information for predicting good functional outcomes and the benefit of EVT. The predictive accuracy of our CT imaging-based model was lower than the established MR PREDICTS model. However, after adding two key clinical characteristics (age and NIHSS) that are almost always available at the point of treatment decision-making, the predictive accuracy became close to MR PREDICTS. Finally, brain frailty imaging features are associated with worse functional outcomes after EVT but do not modify the effect of EVT on functional outcome, nor provide independent added predictive value over clinical and stroke-related imaging characteristics incorporated in MR PREDICTS.

## Supplementary information

Supplementary information

## Abbreviations

ASPECTS: Alberta stroke program early CT score
BPF: Brain parenchymal fraction
EVT: Endovascular thrombectomy
ICAC: Intracranial carotid artery calcification
LVO: Large vessel occlusion
mRS: Modified Rankin scale
NIHSS: National Institutes of Health Stroke Scale
WML: White matter lesion
