# Supplementary material for: A CT imaging-based prediction model of functional outcome and benefit of endovascular thrombectomy for ischemic stroke
Source: Eur Radiol. 2026 Jan 14;36(6):4851–61. doi: 10.1007/s00330-025-12207-7 (PMC13212636; doi:10.1007/s00330-025-12207-7)
Supplement: Supplementary file 1 — Supplementary information [file 330_2025_12207_MOESM1_ESM.pdf]

A CT imaging-based prediction model of functional outcome and benefit of endovascular thrombectomy for ischemic stroke

ELECTRONIC SUPPLEMENTARY MATERIAL

Eur Radiol (2025) Luijten SPR, Ganesh A, Marcus AP, et al.

Image processing

White matter lesions and old infarcts

For segmentation of white matter lesions (WML) and old infarcts, we employed independent U-Net models[1] sharing the same architecture but trained using different datasets. For WML, we used non-contrast CT data of ischemic stroke patients presenting to the Hyperacute Stroke Unit at Imperial College London between 2010 and 2014 as previously described.[2] Automated-delineated WML volumes correlated strongly with expert-delineated WML volumes on MR imaging and on CT (r^2=0.85, 0.71 respectively). Additional evaluation of spatial similarity showed a Dice score of 0.65 between expert and automated-delineated WML.[3] For old infarcts, we used non-contrast CT data of ischemic stroke patients who presented to Northwick Park Hospital between 2013 to 2016. Images were processed using the pipeline recommended by Muschelli[4] and ground truth masks were produced by manual annotation from experts. Automated delineated old infarct volumes correlated strongly with expert-delineated old infarct volumes (r^2=0.82). Evaluation of spatial similarity showed a Dice score of 0.63 and of 0.82 for old infarct volumes >1 milliliter.[3] The models comprised of four levels of typical blocks containing convolutional, pooling or unpooling, and batch normalization layers; and utilized a stack of 15 consecutive slices with an in-plane resolution of 256 x 256 as input. They were trained using 5-fold cross-validation for 100 epochs with a batch size of 128, and used the Adam optimizer[5] with a learning rate of 0.0001 and cross-entropy loss. Presence of old infarct lesions was additionally confirmed by an imaging panel consisting of four experienced raters (2 radiologists, FaBe: 4 years of experience, and IA: 6 years of experience; 1 neuro-radiologist, FB: 7 years of experience; and 1 stroke neurologist, JF: 7 years of experience) who assessed all images for presence of old infarcts while blinded for all patient characteristics including output of the segmentation model. Disagreements between readers were resolved by consensus reading by an additional reader (AG, stroke neurologist with 10 years of experience).

If multiple old infarcts were present on a single scan, the total volume of old infarcts was computed.

Brain atrophy

Brain atrophy was determined as the proportion of total brain volume (TBV) in relation to intracranial volume (ICV) and expressed as the brain parenchymal fraction (BPF, in %). TBV and ICV were computed using an algorithm for segmenting and spatially normalizing non-contrast CT scans (CTseg, code available at https://github.com/WCHN/CTseg).[6] The segmentation algorithm is an extension of the unified segmentation routine implemented in SPM12 with improved image registration and tissue atlases learned from both MRIs and CTs.[7] After skull stripping, the algorithm produces tissue masks of grey matter (GM), white matter (WM), and cerebrospinal fluid (CSF). We computed TBV as the sum of GM and WM, and computed ICV as the sum of GM, WM, and CSF in native space. WM masks included both normal-appearing WM and WML. In patients with old infarcts, care was taken to exclude these from TBV segmentations.

Manual correction of segmentation errors

The quality of all segmentations was visually inspected for inaccuracies by a radiology resident (SL: 4 years of experience).[8; 9] Segmentation errors were manually corrected by adding or removing voxels in three-dimensional orientation within ITKSnap (http://itksnap.org). Examples of WML, TBV, ICV, and old infarct segmentations used for analysis are provided in Supplementary figure 2.

Supplementary table 1. Comparison of brain frailty characteristics per quartile of advancing age

	**1st quartile** (20-57 years)	**2nd quartile** (58-67 years)	**3rd quartile** (68-76 years)	**4th quartile** (77-97 years)	**p-value**
**Any WML**	196 (61.8%)	272 (82.7%)	299 (93.4%)	324 (98.2%)	<0.001
**WML volume** (mL)	0.63 (0.32-1.27)	0.72 (0.31-1.73)	1.22 (0.42-3.09)	1.87 (0.75-4.75)	<0.001
**BPF** (%)	82.00 (2.35)	80.97 (2.40)	79.82 (2.42)	78.74 (2.69)	<0.001
**Any old infarct**	41 (12.8%)	78 (23.6%)	99 (31.0%)	101 (30.3%)	<0.001
**Old infarct volume** (mL)	1.16 (0.17-5.42)	0.55 (0.13-4.45)	0.69 (0.11-6.47)	0.43 (0.12-5.80)	0.80
**ICAC subtype**					<0.001
No calcification	206 (63.0%)	87 (25.7%)	37 (11.2%)	15 (4.4%)	
Intimal	88 (26.9%)	135 (39.8%)	96 (29.0%)	74 (21.9%)	
Medial	33 (10.1%)	117 (34.5%)	198 (59.8%)	249 (73.7%)	

Data are presented as count (%), mean (SD), or median (IQR).
BPF indicates brain parenchymal fraction; WML, white matter lesion; ICAC, intracranial carotid artery calcification.

Supplemental table 2. Univariable association between brain frailty measures and functional outcome in HERMES (n=1391)

	**Common odds ratio (95% CI)**
**WML volume**, per 5 mL	0.71 (0.63-0.79)
**BPF**, per %	1.10 (1.06-1.14)
**Old infarct volume**, per 5 mL	0.95 (0.88-1.02)
**ICAC subtype**	
No calcification	1.00 (reference)
Intimal subtype	0.78 (0.61-1.00)
Medial	0.41 (0.33-0.53)

All effect estimates represent common odds ratios with 95% CIs where an odds ratio >1 corresponds with better functional outcome.
BPF indicates brain parenchymal fraction; WML, white matter lesion; ICAC, intracranial carotid artery calcification.

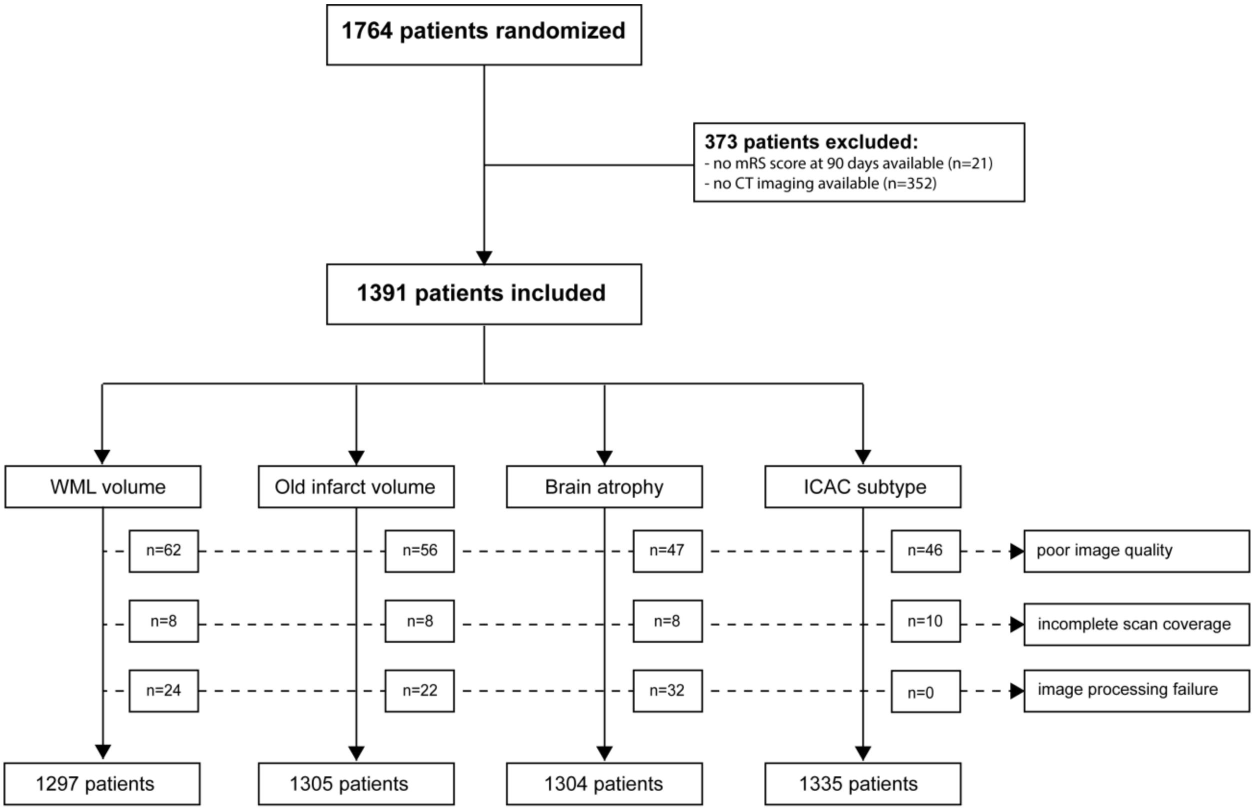

Supplementary figure 1. Flowchart describing total number of patients included in the present analysis (n=1391) and number of patients assessed for different brain frailty features. Patients in whom brain frailty features could not be assessed due to poor image quality, incomplete scan coverage, or image processing failures were included in the analysis and missing data was imputed.

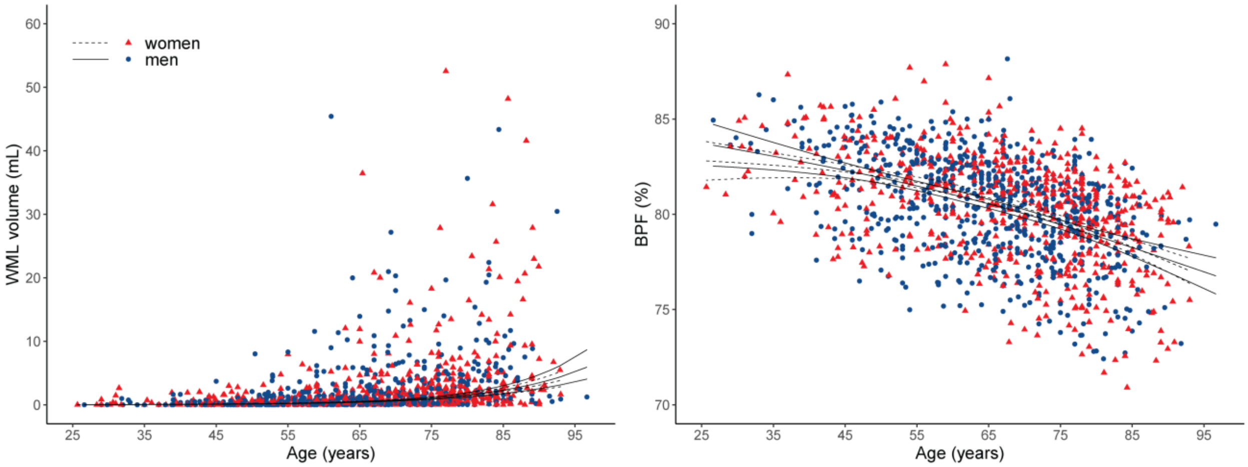

Supplementary figure 2. White matter lesion (WML) volume and brain parenchymal fraction (BPF) versus advancing age with regression lines for nonlinear fit with corresponding 95% confidence intervals. (A) WML trajectories were estimated by first log-transforming WML volumes to account for the skewness of the distribution and then back-transformed to obtain absolute WML volumes. (B) BPF trajectories were estimated using a natural spline of age with 1 knot to account for nonlinear decrease of BPF with advancing age. Data are stratified by sex. Women: red triangles and dashed line. Men: blue circles and solid line.

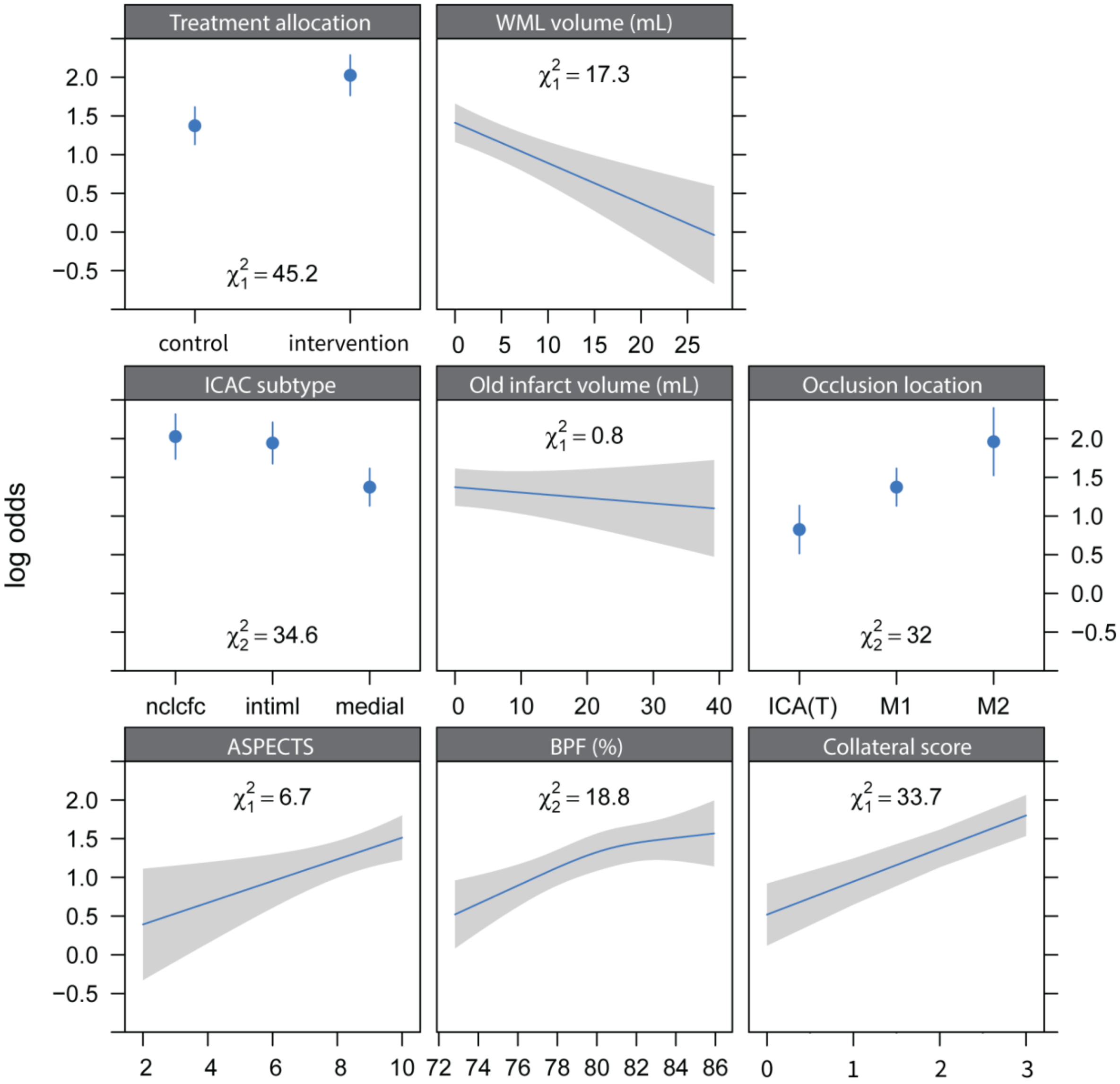

Supplementary figure 3. Effects of variables in CT imaging-based model. The graphs depict the log odds for a shift towards a better mRS score (ordinal scale, 0-6) where a higher log odds corresponds to an increased likelihood of achieving a better functional outcome. The importance of each variable in the CT imaging-based model is indicated using Wald chi-squared values.

WML indicates white matter lesion; nclcfc, no calcification; Intiml, intimal; ICA(T), intracranial carotid artery or ICA terminus; M1, M1 segment of the middle cerebral artery; M2, M2 segment of the middle cerebral artery; ICAC, intracranial carotid artery calcification; ASPECTS, Alberta Stroke Program Early CT Score; BPF, brain parenchymal fraction.

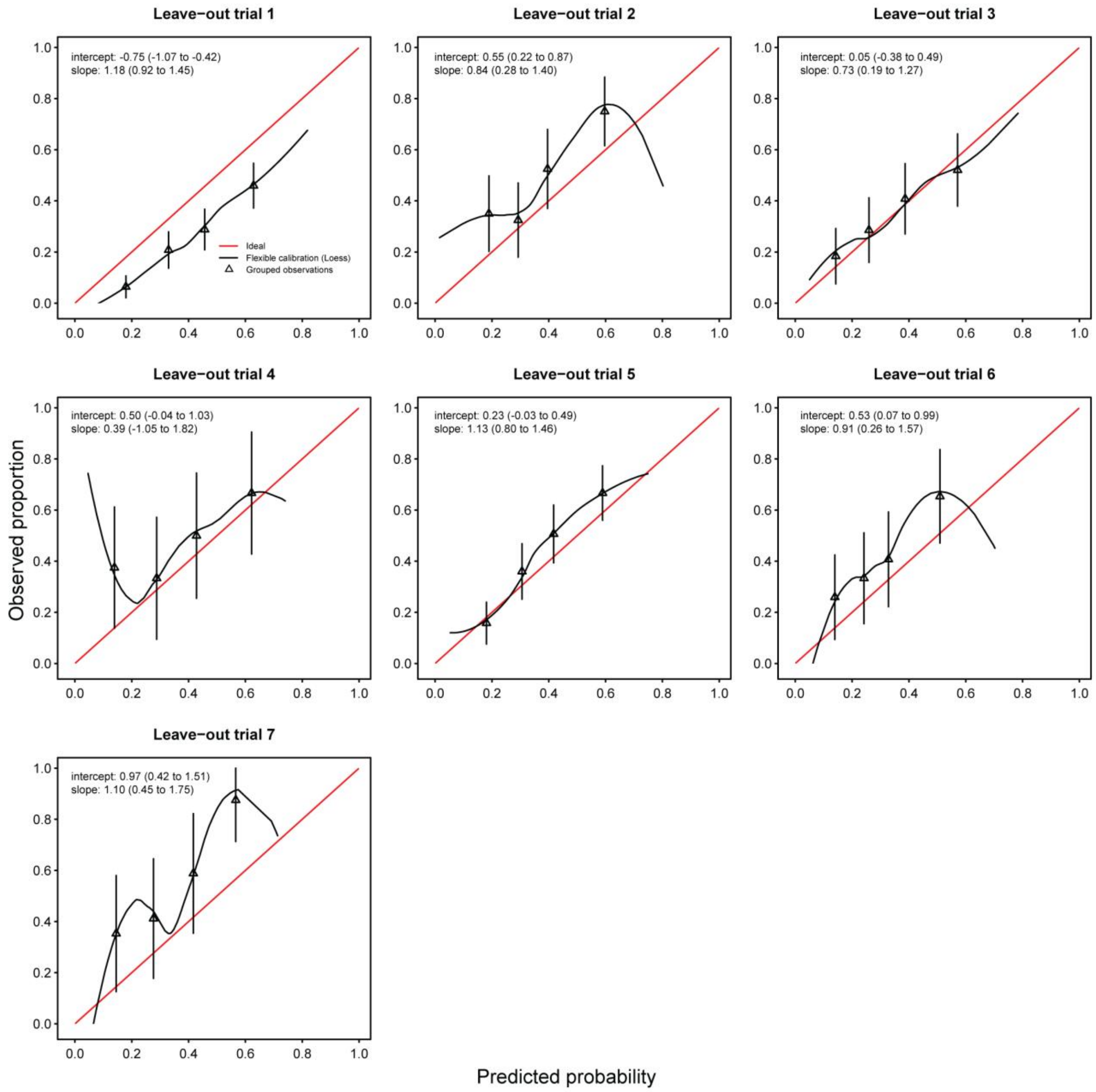

Supplementary figure 4. Calibration plots of CT imaging-based model for prediction of good functional outcome. Model developed and validated in a leave-one-study-out cross-validation. The triangles indicate the actual observed outcomes per quartile of predicted probability.

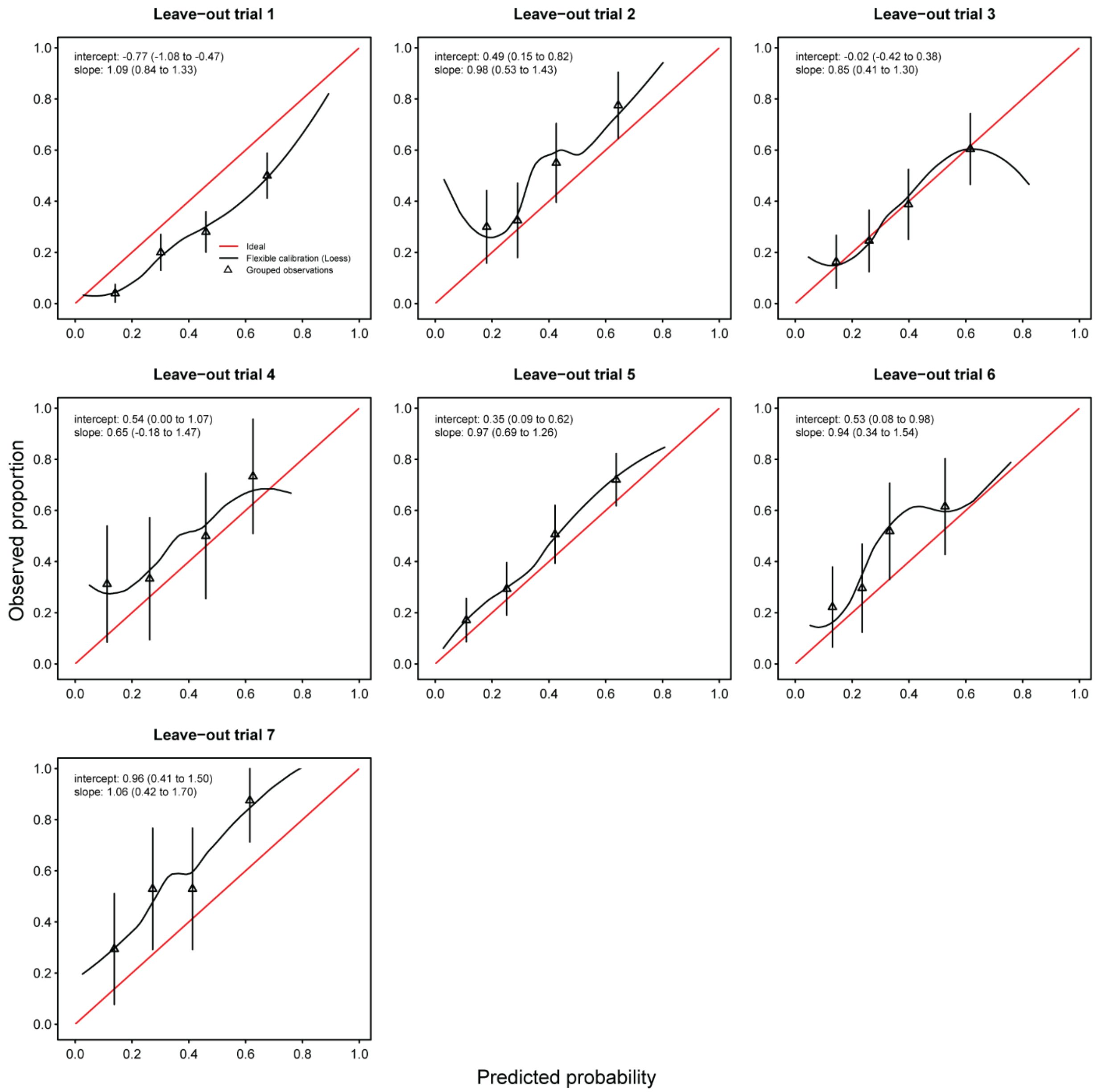

Supplementary Figure 5. Calibration plots of CT imaging-based model extended with age and NIHSS for prediction of good functional outcome. Model developed and validated in a leave-one-study-out cross-validation. The triangles indicate the actual observed outcomes per quartile of predicted probability.

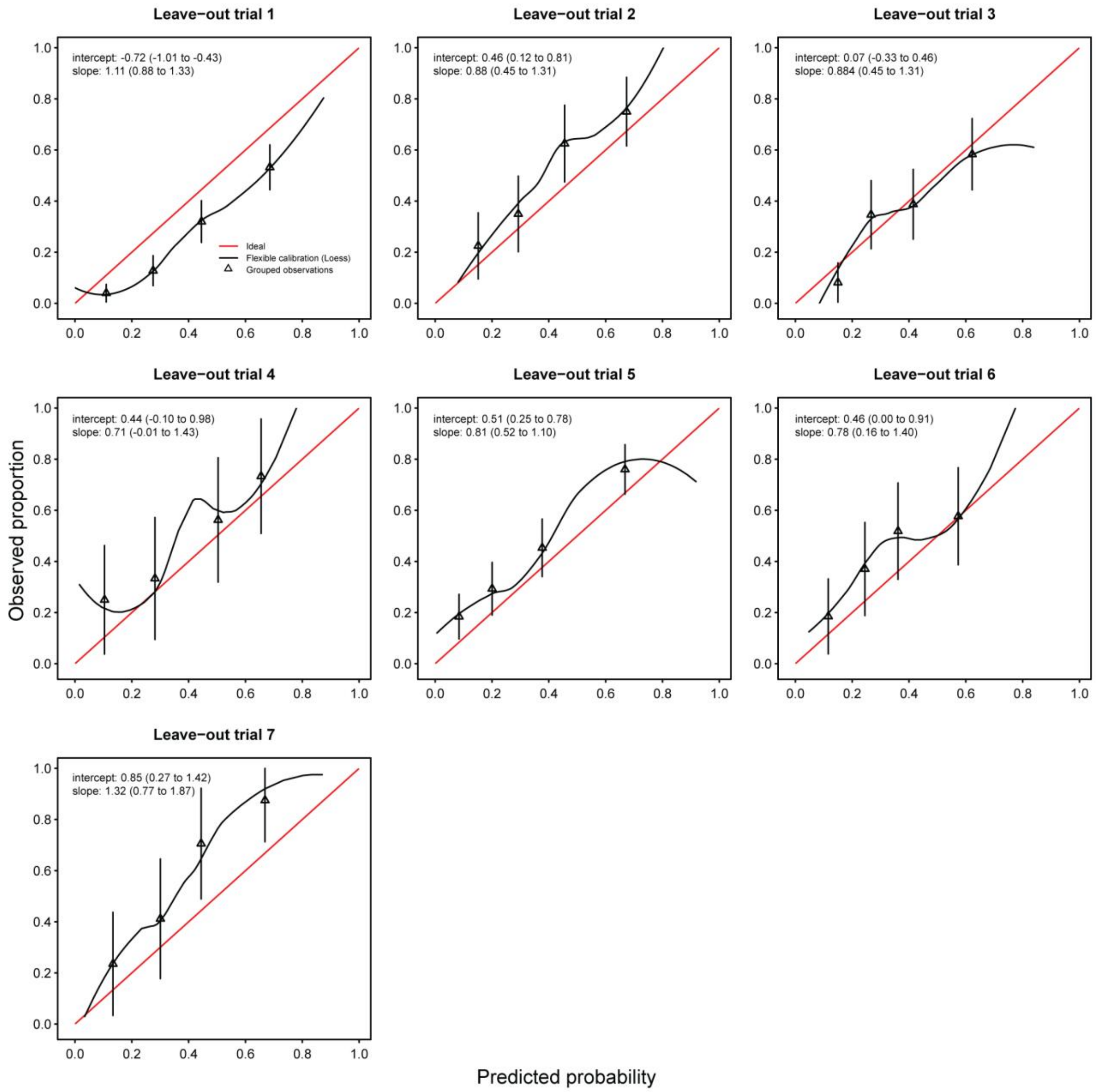

Supplementary figure 6. Calibration plots of MR PREDICTS model for prediction of good functional outcome. Model developed and validated in a leave-one-study-out cross-validation. The triangles indicate the actual observed outcomes per quartile of predicted probability.

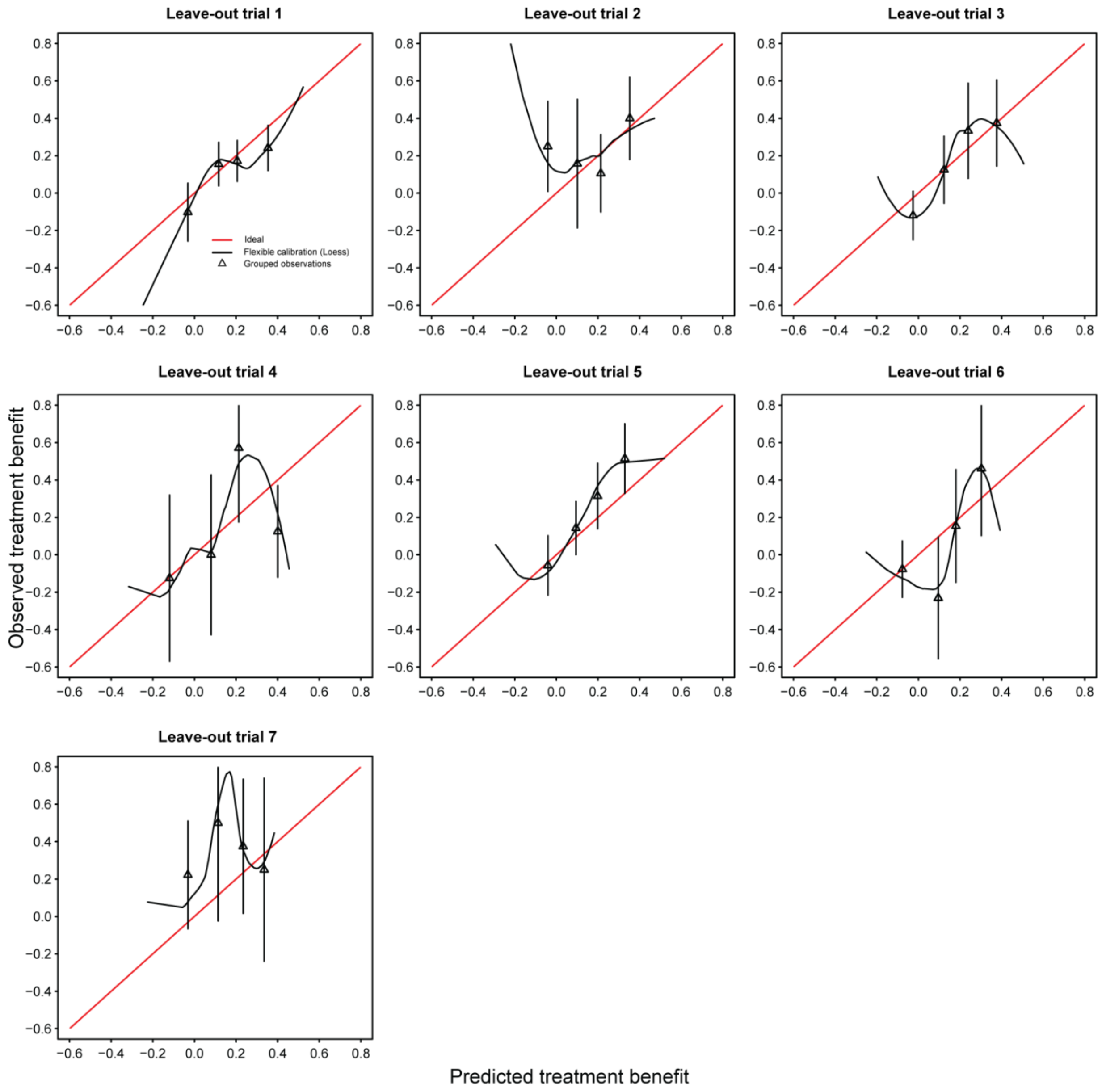

Supplementary figure 7. Calibration plots of CT imaging-based model for predicting treatment benefit. Model developed and validated using leave-one-study-out cross-validation.

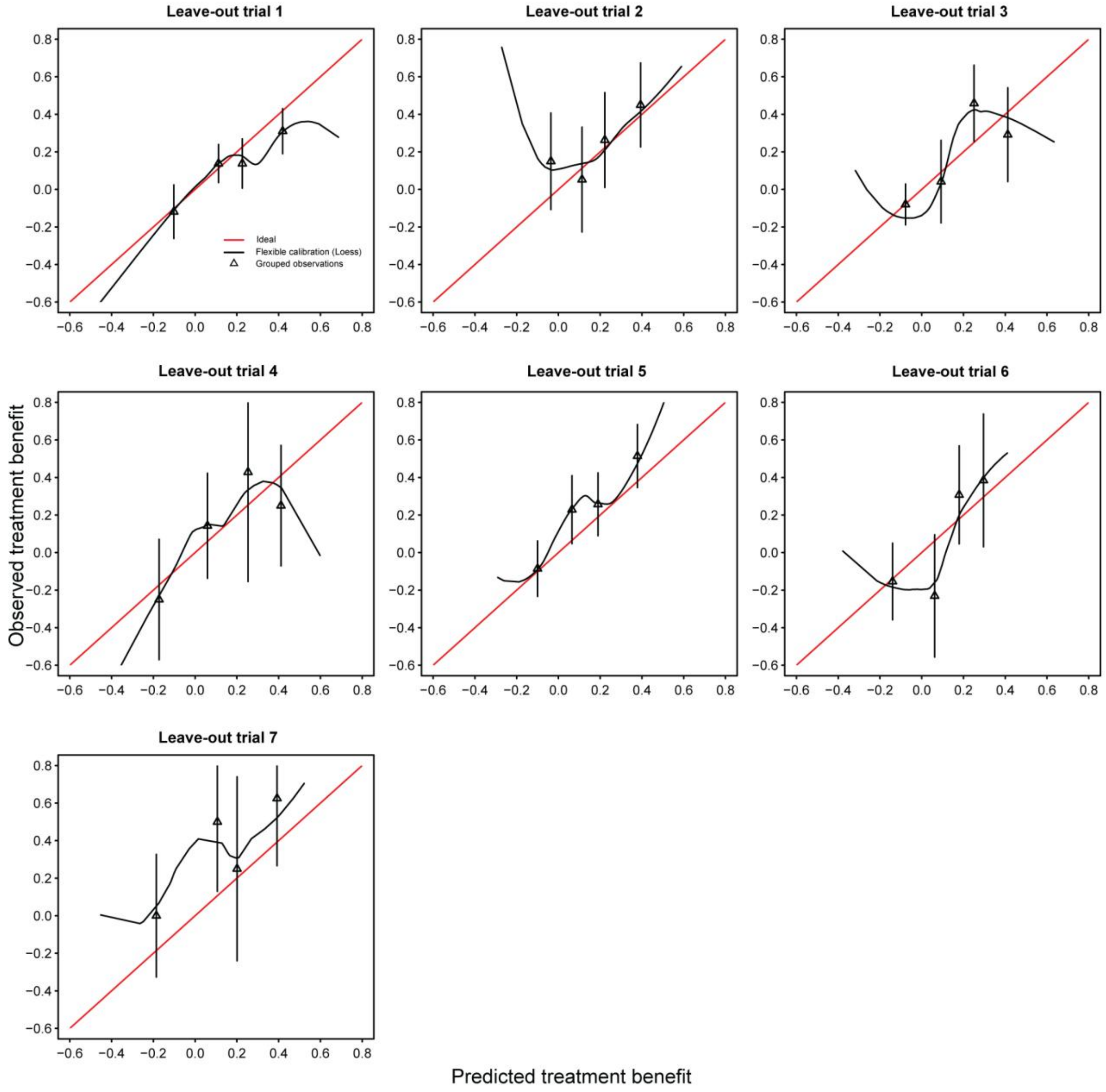

Supplementary figure 8. Calibration plots of CT imaging-based model expanded with age and NIHSS for predicting treatment benefit. Model developed and validated using leave-one-study-out cross-validation.

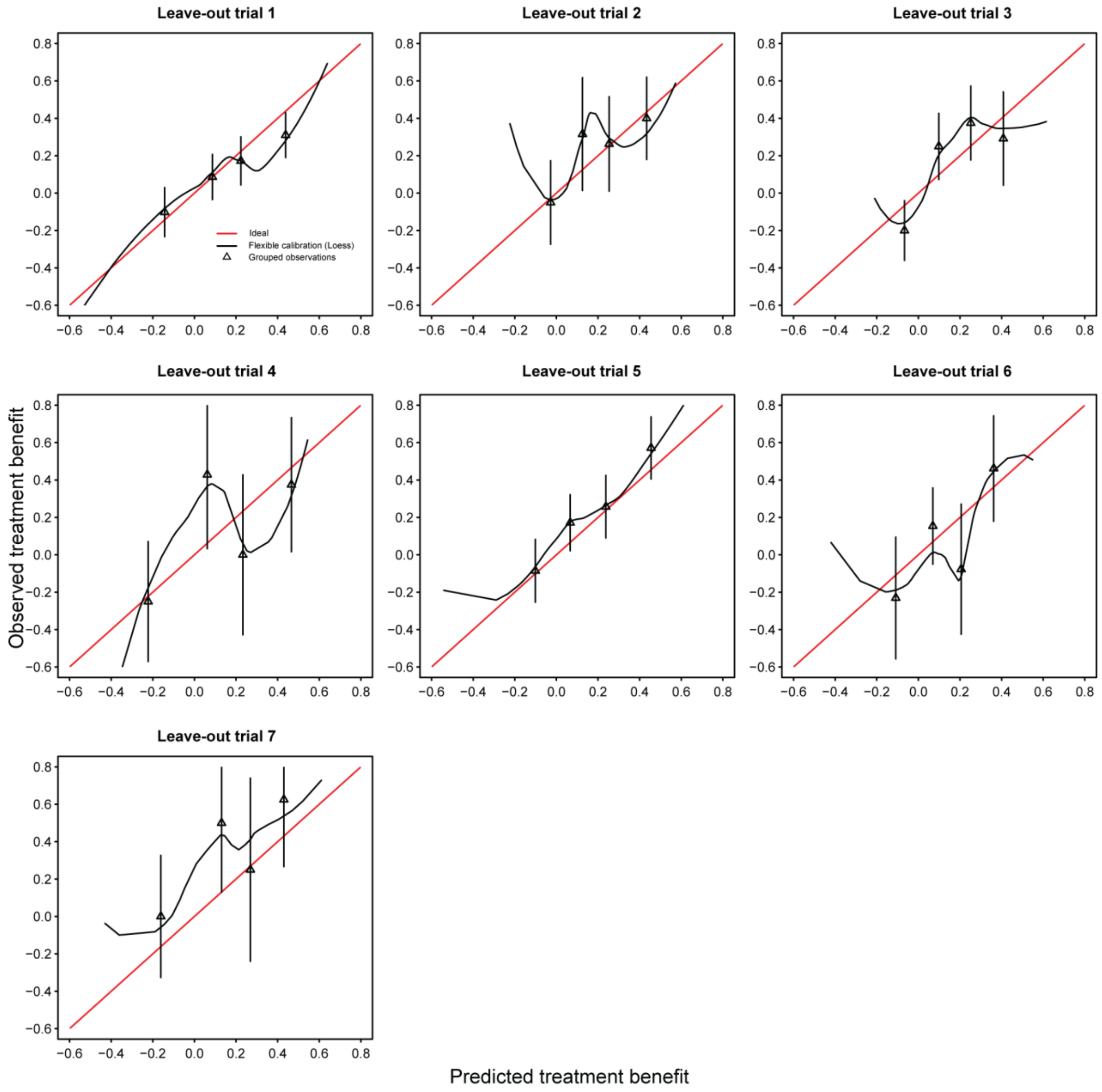

Supplementary figure 9. Calibration plots of MR PREDICTS model for predicting treatment benefit. Model developed and validated using leave-one-study-out cross-validation.

Supplementary references

1 Ronneberger O, Fischer P, Brox T (2015) U-Net: Convolutional Networks for Biomedical Image Segmentation. Springer International Publishing, Cham, pp 234-241

2 Chen L, Carlton Jones AL, Mair G et al (2018) Rapid Automated Quantification of Cerebral Leukoaraiosis on CT Images: A Multicenter Validation Study. Radiology 288:573-581

3 Chen L, Hallet C, Fernandes C et al (2019) Automated Multi-Feature Qauntification of Plain CT in Acute Stroke (Poster). ESOC.

4 Muschelli J (2019) Recommendations for Processing Head CT Data. Front Neuroinform 13:61

5 Kingma DP, Ba JL (2014) Adam: A method for stochastic optimization. In: arXiv:14126980. ap, (ed) Proceedings of the 3rd International Conference on Learning Representations (ICLR),

6 Brudfors M, Balbastre Y, Flandin G, Nachev P, Ashburner J (2020) Flexible Bayesian Modelling for Nonlinear Image RegistrationInternational Conference on Medical Image Computing and Computer-Assisted Intervention. Springer, pp 253-263

7 Ashburner J, Friston KJ (2005) Unified segmentation. Neuroimage 26:839-851

8 Luijten SPR, Bos D, Compagne KCJ et al (2021) Association of White Matter Lesions and Outcome After Endovascular Stroke Treatment. Neurology 96:e333-e342

9 Luijten SP, Compagne KC, van Es AC et al (2021) Brain atrophy and endovascular treatment effect in acute ischemic stroke: a secondary analysis of the MR CLEAN trial. Int J Stroke. 10.1177/17474930211054964:17474930211054964

HERMES Collaborators

Berkhemer OA, Fransen PS, Beumer D, van den Berg LA, Lingsma HF, Yoo AJ, Schonewille WJ, Vos JA, Nederkoorn PJ, Wermer MJ, van Walderveen MA, Staals J, Hofmeijer J, van Oostayen JA, Lycklama À Nijeholt GJ, Boiten J, Brouwer PA, Emmer BJ, de Bruijn SF, van Dijk LC, Kappelle J, Lo RH, van Dijk EJ, de Vries J, de Kort PLM, van Rooij WJJ, van den Berg JSP, van Hasselt BAAM, Aerden LAM, Dallinga RJ, Visser MC, Bot JCJ, Vroomen PC, Eshghi O, Schreuder THCML, Heijboer RJJ, Keizer K, Tielbeek AV, den Hertog HM, Gerrits DG, van den Berg-Vos RM, Karas GB, Steyerberg EW, Flach Z, Marquering HA, Sprengers MES, Jenniskens SFM, Beenen LFM, van den Berg R, Koudstaal PJ, van Zwam WH, Roos YBWEM, van der Lugt A, van Oostenbrugge RJ, Majoie CBLM, Dippel DWJ, Brown MM, Liebig T, Stijnen T, Andersson T, Mattle H, Wahlgren N, van der Heijden E, Ghannouti N, Fleitour N, Hooijenga I, Puppels C, Pellikaan W, Geerling A, Lindl-Velema A, van Vemde G, de Ridder A, Greebe P, de Bont-Stikkelbroeck J, de Meris J, Janssen K, Struijk W, Licher S, Boodt N, Ros A, Venema E, Slokkers I, Ganpat RJ, Mulder M, Saiedie N, Heshmatollah A, Schipperen S, Vinken S, van Boxtel T, Koets J, Boers M, Santos E, Borst J, Jansen I, Kappelhof M, Lucas M, Geuskens R, Barros RS, Dobbe R, Csizmadia M, Hill MD, Goyal M, Demchuk AM, Menon BK, Eesa M, Ryckborst KJ, Wright MR, Kamal NR, Andersen L, Randhawa PA, Stewart T, Patil S, Minhas P, Almekhlafi M, Mishra S, Clement F, Sajobi T, Shuaib A, Montanera WJ, Roy D, Silver FL, Jovin TG, Frei DF, Sapkota B, Rempel JL, Thornton J, Williams D, Tampieri D, Poppe AY, Dowlatshahi D, Wong JH, Mitha AP, Subramaniam S, Hull G, Lowerison MW, Sajobi T, Salluzzi M, Wright MR, Maxwell M, Lacusta S, Drupals E, Armitage K, Barber PA, Smith EE, Morrish WF, Coutts SB, Derdeyn C, Demaerschalk B, Yavagal D, Martin R, Brant R, Yu Y, Willinsky RA, Montanera WJ, Weill A, Kenney C, Aram H, Stewart T, Stys PK, Watson TW, Klein G, Pearson D, Couillard P, Trivedi A, Singh D, Klourfeld E, Imoukhuede O, Nikneshan D, Blayney S, Reddy R, Choi P, Horton M, Musuka T, Dubuc V, Field TS, Desai J, Adatia S, Alseraya A, Nambiar V, van Dijk R, Wong JH, Mitha AP, Morrish WF, Eesa M, Newcommon NJ, Shuaib A, Schwindt B, Butcher KS, Jeerakathil T, Buck B, Khan K, Naik SS, Emery DJ, Owen RJ, Kotylak TB, Ashforth RA, Yeo TA, McNally D, Siddiqui M, Saqqur M, Hussain D, Kalashyan H, Manosalva A, Kate M, Gioia L, Hasan S, Mohammad A, Muratoglu M, Williams D, Thornton J, Cullen A, Brennan P, O'Hare A, Looby S, Hyland D, Duff S, McCusker M, Hallinan B, Lee S, McCormack J, Moore A, O'Connor M, Donegan C, Brewer L, Martin A, Murphy S, O'Rourke K, Smyth S, Kelly P, Lynch T, Daly T, O'Brien P, O'Driscoll A, Martin M, Daly T, Collins R, Coughlan T, McCabe D, Murphy S, O'Neill D, Mulroy M, Lynch O, Walsh T, O'Donnell M, Galvin T, Harbison J, McElwaine P, Mulpeter K, McLoughlin C, Reardon M, Harkin E, Dolan E, Watts M, Cunningham N, Fallon C, Gallagher S, Cotter P, Crowe M, Doyle R, Noone I, Lapierre M, Coté VA, Lanthier S, Odier C, Durocher A, Raymond J, Weill A, Daneault N, Deschaintre Y, Jankowitz B, Baxendell L, Massaro L, Jackson-Graves C, Decesare S, Porter P, Armbruster K, Adams A, Billigan J, Oakley J, Ducruet A, Jadhav A, Giurgiutiu DV, Aghaebrahim A, Reddy V, Hammer M, Starr M,

Totoraitis V, Wechsler L, Streib S, Rangaraju S, Campbell D, Rocha M, Gulati D, Silver FL, Krings T, Kalman L, Cayley A, Williams J, Stewart T, Wiegner R, Casaubon LK, Jaigobin C, Del Campo JM, Elamin E, Schaafsma JD, Willinsky RA, Agid R, Farb R, Ter Brugge K, Sapkoda BL, Baxter BW, Barton K, Knox A, Porter A, Sirelkhatim A, Devlin T, Dellinger C, Pitiyanuvath N, Patterson J, Nichols J, Quarfordt S, Calvert J, Hawk H, Fanale C, Frei DF, Bitner A, Novak A, Huddle D, Bellon R, Loy D, Wagner J, Chang I, Lampe E, Spencer B, Pratt R, Bartt R, Shine S, Dooley G, Nguyen T, Whaley M, McCarthy K, Teitelbaum J, Tampieri D, Poon W, Campbell N, Cortes M, Dowlatshahi D, Lum C, Shamloul R, Robert S, Stotts G, Shamy M, Steffenhagen N, Blacquiere D, Hogan M, AlHazzaa M, Basir G, Lesiuk H, Iancu D, Santos M, Choe H, Weisman DC, Jonczak K, Blue-Schaller A, Shah Q, MacKenzie L, Klein B, Kulandaivel K, Kozak O, Gzesh DJ, Harris LJ, Khoury JS, Mandzia J, Pelz D, Crann S, Fleming L, Hesser K, Beauchamp B, Amato-Marzialli B, Boulton M, Lopez-Ojeda P, Sharma M, Lownie S, Chan R, Swartz R, Howard P, Golob D, Gladstone D, Boyle K, Boulos M, Hopyan J, Yang V, Da Costa L, Holmstedt CA, Turk AS, Navarro R, Jauch E, Ozark S, Turner R, Phillips S, Shankar J, Jarrett J, Gubitz G, Maloney W, Vandorpe R, Schmidt M, Heidenreich J, Hunter G, Kelly M, Whelan R, Peeling L, Burns PA, Hunter A, Wiggam I, Kerr E, Watt M, Fulton A, Gordon P, Rennie I, Flynn P, Smyth G, O'Leary S, Gentile N, Linares G, McNelis P, Erkmen K, Katz P, Azizi A, Weaver M, Jungreis C, Faro S, Shah P, Reimer H, Kalugdan V, Saposnik G, Bharatha A, Li Y, Kostyrko P, Santos M, Marotta T, Montanera W, Sarma D, Selchen D, Spears J, Heo JH, Jeong K, Kim DJ, Kim BM, Kim YD, Song D, Lee KJ, Yoo J, Bang OY, Rho S, Lee J, Jeon P, Kim KH, Cha J, Kim SJ, Ryoo S, Lee MJ, Sohn SI, Kim CH, Ryu HG, Hong JH, Chang HW, Lee CY, Rha J, Davis SM, Donnan GA, Campbell BC, Mitchell PJ, Churilov L, Yan B, Dowling R, Yassi N, Oxley TJ, Wu TY, Silver G, McDonald A, McCoy R, Kleinig TJ, Scroop R, Dewey HM, Simpson M, Brooks M, Coulton B, Krause M, Harrington TJ, Steinfort B, Faulder K, Priglinger M, Day S, Phan T, Chong W, Holt M, Chandra RV, Ma H, Young D, Wong K, Wijeratne T, Tu H, Mackay E, Celestino S, Bladin CF, Loh PS, Gilligan A, Ross Z, Coote S, Frost T, Parsons MW, Miteff F, Levi CR, Ang T, Spratt N, Kaauwai L, Badve M, Rice H, de Villiers L, Barber PA, McGuinness B, Hope A, Moriarty M, Bennett P, Wong A, Coulthard A, Lee A, Jannes J, Field D, Sharma G, Salinas S, Cowley E, Snow B, Kolbe J, Stark R, King J, Macdonnell R, Attia J, D'Este C, Saver JL, Goyal M, Diener HC, Levy EI, Bonafé A, Mendes Pereira V, Jahan R, Albers GW, Cognard C, Cohen DJ, Hacke W, Jansen O, Jovin TG, Mattle HP, Nogueira RG, Siddiqui AH, Yavagal DR, von Kummer R, Smith W, Turjman F, Hamilton S, Chiacchierini R, Amar A, Sanossian N, Loh Y, Devlin T, Baxter B, Hawk H, Sapkota B, Quarfordt S, Sirelkhatim A, Dellinger C, Barton K, Reddy VK, Ducruet A, Jadhav A, Horev A, Giurgiutiu DV, Totoraitis V, Hammer M, Jankowitz B, Wechsler L, Rocha M, Gulati D, Campbell D, Star M, Baxendell L, Oakley J, Siddiqui A, Hopkins LN, Snyder K, Sawyer R, Hall S, Costalat V, Riquelme C, Machi P, Omer E, Arquizan C, Mourand I, Charif M, Ayrignac X, Menjot de Champfleur N, Leboucq N, Gascou G, Moynier M, du Mesnil de Rochemont R, Singer O, Berkefeld J, Foerch C, Lorenz M, Pfeilschifer W, Hattingen E, Wagner M, You SJ, Lescher S, Braun H, Dehkharghani S, Belagaje SR, Anderson A, Lima A, Obideen M, Haussen D, Dharia R, Frankel M, Patel V, Owada K, Saad A, Amerson L, Horn C, Doppelheuer S, Schindler K, Lopes DK, Chen M, Moftakhar R, Anton C, Smreczak M, Carpenter JS, Boo S, Rai A, Roberts T, Tarabishy A, Gutmann L, Brooks C, Brick J, Domico J, Reimann G, Hinrichs K, Becker M, Heiss E, Selle C, Witteler A, Al'Boutros S, Danch MJ, Ranft A, Rohde S, Burg K, Weimar C, Zegarac V, Hartmann C, Schlamann M, Göricke S,

Ringlestein A, Wanke I, Mönninghoff C, Dietzold M, Budzik R, Davis T, Eubank G, Hicks WJ, Pema P, Vora N, Mejilla J, Taylor M, Clark W, Rontal A, Fields J, Peterson B, Nesbit G, Lutsep H, Bozorgchami H, Priest R, Ologuntoye O, Barnwell S, Dogan A, Herrick K, Takahasi C, Beadell N, Brown B, Jamieson S, Hussain MS, Russman A, Hui F, Wisco D, Uchino K, Khawaja Z, Katzan I, Toth G, Cheng Ching E, Bain M, Man S, Farrag A, George P, John S, Shankar L, Drofa A, Dahlgren R, Bauer A, Itreat A, Taqui A, Cerejo R, Richmond A, Ringleb P, Bendszus M, Möhlenbruch M, Reiff T, Amiri H, Purrucker J, Herweh C, Pham M, Menn O, Ludwig I, Acosta I, Villar C, Morgan W, Sombutmai C, Hellinger F, Allen E, Bellew M, Gandhi R, Bonwit E, Aly J, Ecker RD, Seder D, Morris J, Skaletsky M, Belden J, Baker C, Connolly LS, Papanagiotou P, Roth C, Kastrup A, Politi M, Brunner F, Alexandrou M, Merdivan H, Ramsey C, Given Ii C, Renfrow S, Deshmukh V, Sasadeusz K, Vincent F, Thiesing JT, Putnam J, Bhatt A, Kansara A, Caceves D, Lowenkopf T, Yanase L, Zurasky J, Dancer S, Freeman B, Scheibe Mirek T, Robison J, Rontal A, Roll J, Clark D, Rodriguez M, Fitzsimmons BF, Zaidat O, Lynch JR, Lazzaro M, Larson T, Padmore L, Das E, Farrow Schmidt A, Hassan A, Tekle W, Cate C, Jansen O, Cnyrim C, Wodarg F, Wiese C, Binder A, Riedel C, Rohr A, Lang N, Laufs H, Krieter S, Remonda L, Diepers M, Añon J, Nedeltchev K, Kahles T, Biethahn S, Lindner M, Chang V, Gächter C, Esperon C, Guglielmetti M, Arenillas Lara JF, Martínez Galdámez M, Calleja Sanz AI, Cortijo Garcia E, Garcia Bermejo P, Perez S, Mulero Carrillo P, Crespo Vallejo E, Ruiz Piñero M, Lopez Mesonero L, Reyes Muñoz FJ, Brekenfeld C, Buhk JH, Kruützelmann A, Thomalla G, Cheng B, Beck C, Hoppe J, Goebell E, Holst B, Grzyska U, Wortmann G, Starkman S, Duckwiler G, Jahan R, Rao N, Sheth S, Ng K, Noorian A, Szeder V, Nour M, McManus M, Huang J, Tarpley J, Tateshima S, Gonzalez N, Ali L, Liebeskind D, Hinman J, Calderon Arnulphi M, Liang C, Guzy J, Koch S, DeSousa K, Gordon Perue G, Haussen D, Elhammady M, Peterson E, Pandey V, Dharmadhikari S, Khandelwal P, Malik A, Pafford R, Gonzalez P, Ramdas K, Andersen G, Damgaard D, Von Weitzel Mudersbach P, Simonsen C, Ruiz de Morales Ayudarte N, Poulsen M, Sørensen L, Karabegovich S, Hjørringgaard M, Hjort N, Harbo T, Sørensen K, Deshaies E, Padalino D, Swarnkar A, Latorre JG, Elnour E, El Zammar Z, Villwock M, Farid H, Balgude A, Cross L, Hansen K, Holtmannspötter M, Kondziella D, Hoejgaard J, Taudorf S, Soendergaard H, Wagner A, Cronquist M, Stavngaard T, Cortsen M, Krarup LH, Hyldal T, Haring HP, Guggenberger S, Hamberger M, Trenkler J, Sonnberger M, Nussbaumer K, Dominger C, Bach E, Jagadeesan BD, Taylor R, Kim J, Shea K, Tummala R, Zacharatos H, Sandhu D, Ezzeddine M, Grande A, Hildebrandt D, Miller K, Scherber J, Hendrickson A, Jumaa M, Zaidi S, Hendrickson T, Snyder V, Killer Oberpfalzer M, Mutzenbach J, Weymayr F, Broussalis E, Stadler K, Jedlitschka A, Malek A, Mueller Kronast N, Beck P, Martin C, Summers D, Day J, Bettinger I, Holloway W, Olds K, Arkin S, Akhtar N, Boutwell C, Crandall S, Schwartzman M, Weinstein C, Brion B, Prothmann S, Kleine J, Kreiser K, Boeckh Behrens T, Poppert H, Wunderlich S, Koch ML, Biberacher V, Huberle A, Gora Stahlberg G, Knier B, Meindl T, Utpadel Fischler D, Zech M, Kowarik M, Seifert C, Schwaiger B, Puri A, Hou S, Wakhloo A, Moonis M, Henniger N, Goddeau R, Massari F, Minaeian A, Lozano JD, Ramzan M, Stout C, Patel A, Tunguturi A, Onteddu S, Carandang R, Howk M, Ribó M, Sanjuan E, Rubiera M, Pagola J, Flores A, Muchada M, Meler P, Huerga E, Gelabert S, Coscojuela P, Tomasello A, Rodriguez D, Santamarina E, Maisterra O, Boned S, Seró L, Rovira A, Molina CA, Millán M, Muñoz L, Pérez de la Ossa N, Gomis M, Dorado L, López-Cancio E, Palomeras E, Munuera J, García Bermejo P, Remollo S, Castaño C, García-Sort R, Cuadras P, Puyalto P,

Hernández-Pérez M, Jiménez M, Martínez-Piñeiro A, Lucente G, Dávalos A, Chamorro A, Urra X, Obach V, Cervera A, Amaro S, Llull L, Codas J, Balasa M, Navarro J, Ariño H, Aceituno A, Rudilosso S, Renu A, Macho JM, San Roman L, Blasco J, López A, Macías N, Cardona P, Quesada H, Rubio F, Cano L, Lara B, de Miquel MA, Aja L, Serena J, Cobo E, Albers GW, Lees KR, Arenillas J, Roberts R, Minhas P, Al-Ajlan F, Salluzzi M, Zimmel L, Patel S, Eesa M, Martí-Fàbregas J, Jankowitz B, Serena J, Salvat-Plana M, López-Cancio E, Bracard S, Ducrocq X, Anxionnat R, Baillot PA, Barbier C, Derelle AL, Lacour JC, Richard S, Samson Y, Sourour N, BaronnetChauvet F, Clarencon F, Crozier S, Deltour S, Di Maria F, Le Bouc R, Leger A, Mutlu G, Rosso C, Szatmary Z, Yger M, Zavanone C, Bakchine S, Pierot L, Caucheteux N, Estrade L, Kadziolka K, Leautaud A, Renkes C, Serre I, Desal H, Guillon B, Boutoleau-Bretonniere C, Daumas-Duport B, De Gaalon S, Derkinderen P, Evain S, Herisson F, Laplaud DA, Lebouvier T, Lintia-Gaultier A, PoucletCourtemanche H, Rouaud T, Rouaud Jaffrenou V, Schunck A, Sevin-Allouet M, Toulgoat F, Wiertlewski S, Gauvrit JY, Ronziere T, Cahagne V, Ferre JC, Pinel JF, Raoult H, Mas JL, Meder JF, Al NajjarCarpentier AA, Birchenall J, Bodiguel E, Calvet D, Domigo V, Godon-Hardy S, Guiraud V, Lamy C, Majhadi L, Morin L, Naggara O, Trystram D, Turc G, Berge J, Sibon I, Menegon P, Barreau X, Rouanet F, Debruxelles S, Kazadi A, Renou P, Fleury O, Pasco-Papon A, Dubas F, Caroff J, Godard Ducceschi S, Hamon MA, Lecluse A, Marc G, Giroud M, Ricolfi F, Bejot Y, Chavent A, Gentil A, Kazemi A, Osseby GV, Voguet C, Mahagne MH, Sedat J, Chau Y, Suissa L, Lachaud S, Houdart E, Stapf C, Buffon Porcher F, Chabriat H, Guedin P, Herve D, Jouvent E, Mawet J, Saint-Maurice JP, Schneble HM, Turjman F, Nighoghossian N, Berhoune NN, Bouhour F, Cho TH, Derex L, Felix S, Gervais-Bernard H, Gory B, Manera L, Mechtouff L, Ritzenthaler T, Riva R, Salaris Silvio F, Tilikete C, Blanc R, Obadia M, Bartolini MB, Gueguen A, Piotin M, Pistocchi S, Redjem H, Drouineau J, Neau JP, Godeneche G, Lamy M, Marsac E, Velasco S, Clavelou P, Chabert E, Bourgois N, Cornut-Chauvinc C, Ferrier A, Gabrillargues J, Jean B, Marques AR, Vitello N, Detante O, Barbieux M, Boubagra K, Favre Wiki I, Garambois K, Tahon F, Ashok V, Voguet C, Coskun O, Guedin P, Rodesch G, Lapergue B, Bourdain F, Evrard S, Graveleau P, Decroix JP, Wang A, Sellal F, Ahle G, Carelli G, Dugay MH, Gaultier C, Lebedinsky AP, Lita L, Musacchio RM, Renglewicz-Destuynder C, Tournade A, Vuillemet F, Montoro FM, Mounayer C, Faugeras F, Gimenez L, Labach C, Lautrette G, Denier C, Saliou G, Chassin O, Dussaule C, Melki E, Ozanne A, Puccinelli F, Sachet M, Sarov M, Bonneville JF, Moulin T, Biondi A, De Bustos Medeiros E, Vuillier F, Courtheoux P, Viader F, Apoil-Brissard M, Bataille M, Bonnet AL, Cogez J, Kazemi A, Touze E, Leclerc X, Leys D, Aggour M, Aguettaz P, Bodenant M, Cordonnier C, Deplanque D, Girot M, Henon H, Kalsoum E, Lucas C, Pruvo JP, Zuniga P, Bonafé A, Arquizan C, Costalat V, Machi P, Mourand I, Riquelme C, Bounolleau P, Arteaga C, Faivre A, Bintner M, Tournebize P, Charlin C, Darcel F, Gauthier-Lasalarie P, Jeremenko M, Mouton S, Zerlauth JB, Lamy C, Hervé D, Hassan H, Gaston A, Barral FG, Garnier P, Beaujeux R, Wolff V, Herbreteau D, Debiais S, Murray A, Ford G, Muir KW, White P, Brown MM, Clifton A, Freeman J, Ford I, Markus H, Wardlaw J, Lees KR, Molyneux A, Robinson T, Lewis S, Norrie J, Robertson F, Perry R, Dixit A, Cloud G, Clifton A, Madigan J, Roffe C, Nayak S, Lobotesis K, Smith C, Herwadkar A, Kandasamy N, Goddard T, Bamford J, Subramanian G, Lenthall R, Littleton E, Lamin S, Storey K, Ghatala R, Banaras A, Aeron-Thomas J, Hazel B, Maguire H, Veraque E, Harrison L, Keshvara R, Cunningham J.
